# Multinuclear absolute magnetic resonance thermometry

**DOI:** 10.1038/s42005-019-0252-3

**Published:** 2019-11-29

**Authors:** Emilia V. Silletta, Alexej Jerschow, Guillaume Madelin, Leeor Alon

**Affiliations:** 1 New York University, Department of Chemistry, 100 Washington Square E, New York, NY 10003, USA; 2 Universidad Nacional de Córdoba, Facultad de Matemática, Astronomía, Física y Computación, Medina Allende s/n, X5000HUA Córdoba, Argentina; 3 Instituto de Física Enrique Gaviola, CONICET, Medina Allende s/n, X5000HUA Córdoba, Argentina; 4 New York University School of Medicine, Department of Radiology, Center for Biomedical Imaging, 660 First Avenue, New York, NY 10016, USA.

## Abstract

Non-invasive measurement of absolute temperature is important for proper characterization of various pathologies and for evaluation of thermal dose during interventional procedures. The proton (hydrogen nucleus) magnetic resonance (MR) frequency shift method can be used to map relative temperature changes. However, spatiotemporal variations in the main magnetic field and the lack of local internal frequency reference challenge the determination of absolute temperature. Here, we introduce a multinuclear method for absolute MR thermometry, based on the fact that the hydrogen and sodium nuclei exhibit a unique and distinct characteristic frequency dependence with temperature and with electrolyte concentration. A one-to-one mapping between the precession frequency difference of the two nuclei and absolute temperature is demonstrated. Proof-of-concept experiments were conducted in aqueous solutions with different NaCl concentrations, in agarose gel samples, and in freshly excised ex vivo mouse tissues. One-dimensional chemical shift imaging experiments also demonstrated excellent agreement with infrared measurements.

Magnetic resonance imaging (MRI) has become a valuable diagnostic tool for visualization of subtle pathologies with millimeter resolution. In recent years there has been a growing interest in the utilization of MR techniques to measure temperature changes in vivo^1^. While most MR contrast mechanisms vary with temperature change^2–8^, it has been shown that the proton (^1^H) resonance frequency (PRF) method has the highest sensitivity to thermal change in most tissues^9^. The temperature dependence of the PRF was first discovered by Hindman when conducting nuclear magnetic resonance (NMR) experiments on intermolecular forces and hydrogen bond formation^9^, and adapted to estimate temperature change through MR phase imaging measurements by Ishihara et al.^10^ and De Poorter et al.^11^. The method is currently the gold standard for mapping thermal changes in interventional applications, such as high-intensity focused ultrasound (HIFU)^12–14^, radiofrequency (RF) hyperthermia^15^, RF ablation^16^, and RF power deposition from wireless devices^17^.

The PRF method relies on the subtraction of pre- and post-exposure phase images, or on the local determination of the frequency shift of protons with MR spectroscopy (MRS), to calculate temperature change due to exposure conditions^18,19^, knowing that the chemical shift temperature dependence of proton is approximately −0.01 ppm/°C^20^ in human tissues. However, non-thermal *B*_0_ changes, such as due to movement^21,22^, magnet field drift^23^, flow^23^, or shim changes, greatly limit the applicability of the PRF method. Today, PRF thermometry is restricted to experiments with large thermal gradients or phantom studies with minimal *B*_0_ drift throughout the experiment. Furthermore, PRF methodologies are not capable of reconstruction of absolute temperature in tissues, because an internal frequency reference (in each voxel) is required. Knowledge of the absolute temperature in tissues is particularly important due to the correlation of many pathologies with thermal disruption and is fundamental for quantification of thermal dose during interventional procedures^24–29^. In NMR experiments, internally referenced measurements of absolute temperature are widely used to monitor temperature of samples by measuring the chemical shift between two or more temperature-dependent peaks such as between the OH and CH_2_ groups in ethylene glycol^30^. Internally referenced experiments are robust against instabilities of *B*_0_ because changes in macroscopic *B*_0_ equally shift the independent peaks^31,32^, enabling the reconstruction of absolute temperature. In the brain, the amid proton in N-acetylaspartate (NAA) peak has been utilized as a temperature-independent reference. However, due to the low concentration of NAA in the brain (~10 mmol/L)^33^, challenges associated with water suppression, pH-dependent separation of the NAA-water peaks, and imaging time required to obtain adequate signal-to-noise ratio (SNR), absolute thermometry via imaging of the NAA peak remains challenging^34^.

Fat, which has a chemical shift of 3.5 ppm from the water peak, can also be used as a reference peak for absolute temperature measurement in vivo^23^. Fat contains relatively few hydrogen bonds, and its PRF thermal coefficient is mainly dictated by the volume magnetic susceptibility, which is small compared to that of water^23^. Studies have shown that fat in surrounding tissues can be used to estimate the background *B*_0_ changes^35^, and information provided by the fat peak has been used to improve the temperature change reconstruction. Nonetheless, fat is absent from most organs and does not provide an internal reference of sufficient sensitivity^35,36^. Moreover, even in tissues that contain fat, it has been shown that the average standard deviation of the distribution of water-fat frequency differences within the breast is around ±0.14 ppm and corresponds to an uncertainty of ±14 °C in temperature measurements^37^. A more recent work^38^ also demonstrated that the water-fat frequency difference method can lead to considerable errors in absolute temperature calculation due to the spatial distribution and heterogeneity of water and fat spins within a voxel.

More recently, a new multinuclear method of absolute MR thermometry based on ^129^Xe and ^1^H MR spectroscopic imaging was proposed^39^. Temperature changes can be measured using lipid-dissolved xenon (LDX) in fat, which has a highly sensitive chemical shift temperature dependence of −0.21 ppm/°C (compared to −0.01 ppm/°C for ^1^H in water)^40^. According to the LDX method^39^, absolute temperature can be estimated using the chemical shift of nearby methylene protons as a fixed reference placed at 1.3 ppm from a fictitious ^1^H center frequency. However, the use of methylene protons degrades the accuracy of the PRF method as microscopic susceptibility variations affect lipid and water spins differently. The LDX method is thus limited to tissues or samples with fat, and necessitates the dissolution of ^129^Xe in adipose tissue through xenon gas inhalation, which can be challenging for in vivo experiments, or for samples without lipids.

In this work, we introduce a novel multinuclear approach for absolute MR thermometry based on two endogenous types of molecules in biological tissues, water and sodium ions Na^+^, as well as a general framework for absolute MR thermometry that can be used with any pair of nuclei. We demonstrate that ^23^Na nuclei exhibit an NMR frequency shift dependency with temperature that is roughly twice that of the ^1^H nuclei. Thus, measuring the difference of NMR frequencies of the ^23^Na and ^1^H nuclei provides a one-to-one mapping with temperature, allowing absolute temperature reconstruction with reduced sensitivity to macroscopic *B*_0_ inhomogeneities (or random shim variations), and without the need of a fixed temperature-independent reference peak. Proof-of-concept experiments were conducted in aqueous solutions with different NaCl concentrations, in agarose gel samples, and in freshly-excised ex vivo mouse tissues. One-dimensional chemical shift imaging (CSI) was also performed for two steady-state temperature regimes.

## Results

### Calibration of Δ*α* and Δ*σ*_0_ in solutions

**Fig. 1:**
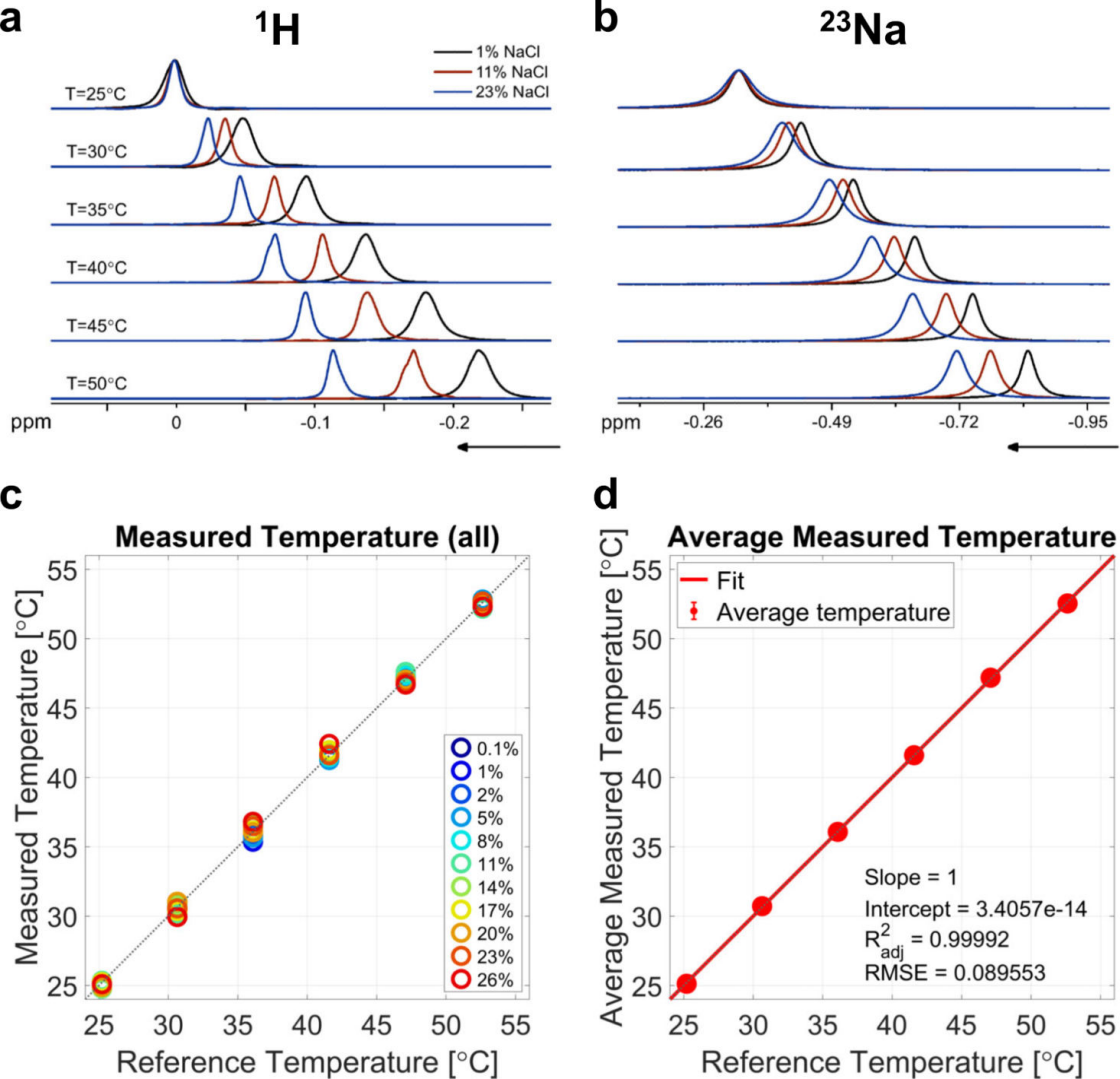
Examples of ^1^H spectra and ^23^Na spectra and temperature measurements. **a** ^1^H and **b** ^23^Na spectra at 6 different temperatures for 3 solutions with different NaCl concentrations: 1, 11, and 23% weight (ppm: parts per million). **c** Temperature measurements in all the calibration samples containing 0.1–26% NaCl, using frequency shift thermal coefficient difference Δ*α* and intercept difference Δ*σ*_0_ calculated from the results shown in Fig. 2, as a function of reference temperatures. **d** Average temperature measurements over all samples as a function of reference temperatures. All temperatures are in °C. Error bars in **d** are small and are included within the data solid circles. $R_{\text{adj}}^{2}$ adjusted *R*^2^, RMSE root mean square error.

We first measured frequency shift thermal coefficient difference Δ*α* and intercept difference Δ*σ*_0_ (see Materials and methods for definitions and calculations) in 11 samples with NaCl concentrations ranging from 0.1 to 26% (saturation) by weight. For each solution, NMR spectra were acquired at 6 different temperatures, as measured by the spectrometer sensor: 25, 30, 35, 40, 45, and 50 °C. The corresponding real temperatures corrected using the spectrometer temperature calibration are shown in Supplementary Note 1 and Supplementary Table 1. The position of the peak maximum followed a linear trend with temperature, with the slope corresponding to the frequency shift thermal coefficient *α*, and the intercept *σ*_0_. Figure 1a, b shows examples of ^1^H and ^23^Na spectra at different temperatures, where the frequency changes with temperature are shown to vary with the NaCl concentration. The lineshapes appear broadened towards higher temperatures as a result of a slight temperature gradient across the sample, as well as probable heating of the shim coils that can alter the magnetic field in the volume of interest. Figure 1c shows the measured temperature for all NaCl solutions after calibration of Δ*α* and Δ*σ*_0_, compared to the reference temperature at which the experiments were performed. Absolute temperatures were calculated using Eq. (12) in Materials and methods. The average measured temperature for all solutions is plotted in Fig. 1d, showing excellent agreement with reference temperature (adjusted $R_{\text{adj}}^{2}=0.99992$, and root mean square error RMSE = 0.09 °C).

**Fig. 2:**
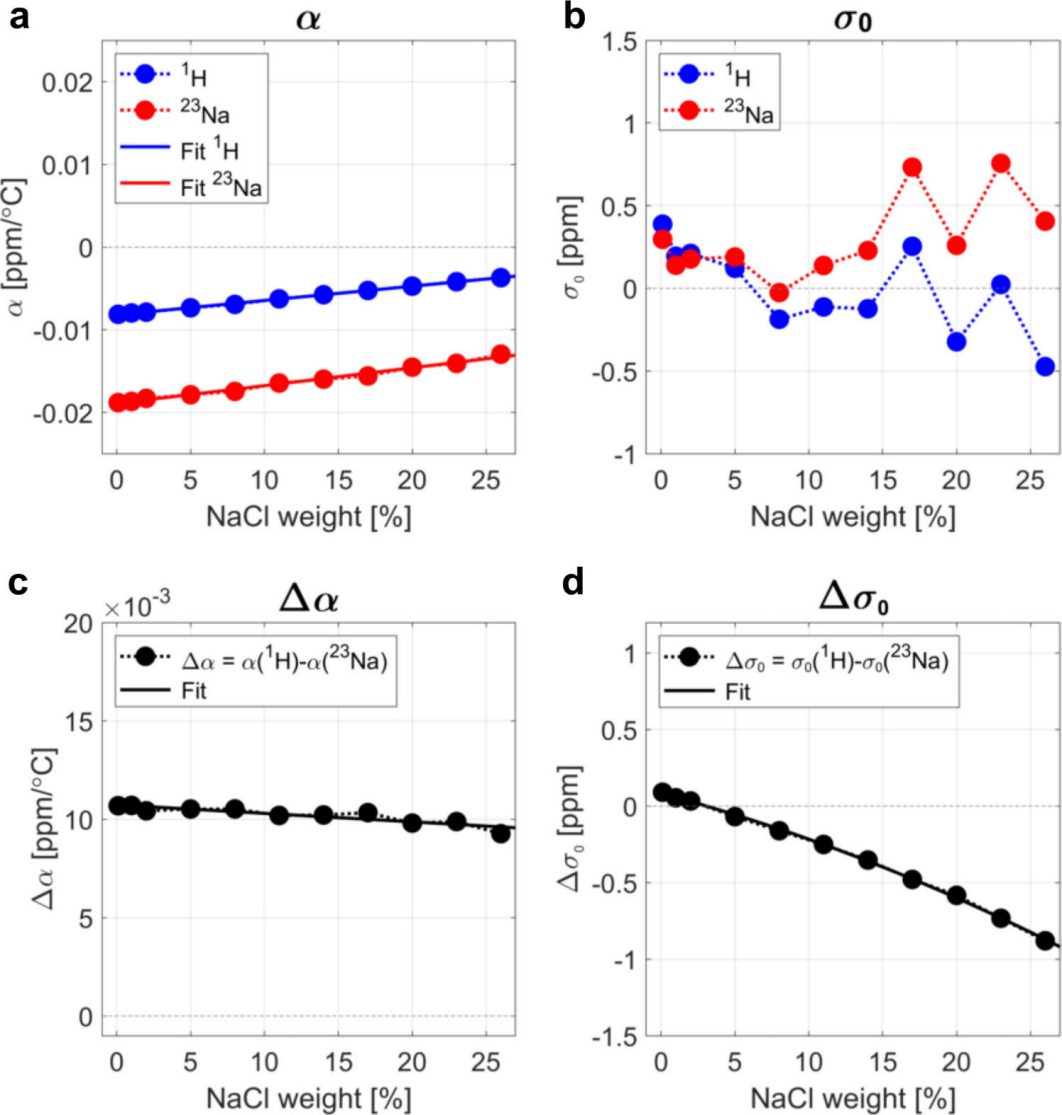
Frequency shift thermal coefficients and intercepts. **a** ^1^H and ^23^Na frequency shift thermal coefficients *α* (slope of the linear fit) at different NaCl concentrations (in % of weight). **b** ^1^H and ^23^Na constants *σ*_0_ (intercept of the linear fit) at different NaCl concentrations (in % of weight). **c** Frequency shift thermal coefficient difference Δ*α* = *α*(^1^H)−*α*(^23^Na) calculated from (**a**), at different NaCl concentrations (in % of weight). **d** Intercept difference Δ*σ*_0_ = *σ*_0_(^1^H)−*σ*_0_(^23^Na) calculated from (**b**), at different NaCl concentrations (in % of weight).

Figure 2 shows the results of the linear fitting of the frequency shift of ^1^H and ^23^Na versus temperature for the 11 samples. The fits are shown in Supplementary Figs. 1–11. The frequency shift thermal coefficient *α* for ^1^H, shown in Fig. 2a, is consistent with literature, where the value of approximately −0.01 ppm/°C is typically found for low NaCl concentrations (1% weight or less in biological tissues). It was found that the frequency shift thermal coefficient *α* for ^23^Na was approximately twice higher in magnitude than for ^1^H. The ^1^H and ^23^Na spectra for each sample were acquired on the same day at six temperatures, and the same shim was used for both nuclei. Different samples were acquired on different days in the following order: 1, 26, 11, 17, 23, 8, 0.1, 2, 5, 14, and 20%. This random order ensures that the smooth variation that was detected for Δ*α* and Δ*σ*_0_ with NaCl concentrations was not an effect of the spectrometer magnetic field drift or *B*_0_ shim changes on different days. These variations of the magnetic field can for example be detected on individual ^1^H and ^23^Na measurements of *σ*_0_ in Fig. 2b. As shown on Fig. 2c, d, both Δ*α* and Δ*σ*_0_ showed a smooth variation with NaCl concentration, even when individual *σ*_0_ values for ^1^H and ^23^Na seem to fluctuate randomly in different samples acquired on different days. The variation of Δ*α* is linear with increased NaCl concentration, while the variation of Δ*σ*_0_ shows a nearly linear decrease with increasing NaCl concentration.

### Effect of pH

In order to study the effect of pH on the multinuclear MR temperature measurements, solutions with different pH values were tested for Δ*α* and Δ*σ*_0_ calibration. The pH range was from 4.9 to 9.07. The results are shown in Supplementary Fig. 12 and demonstrate that pH has negligible influence on the Δ*α* and Δ*σ*_0_ values.

### Blind experiments in 1% NaCl solution

**Fig. 3:**
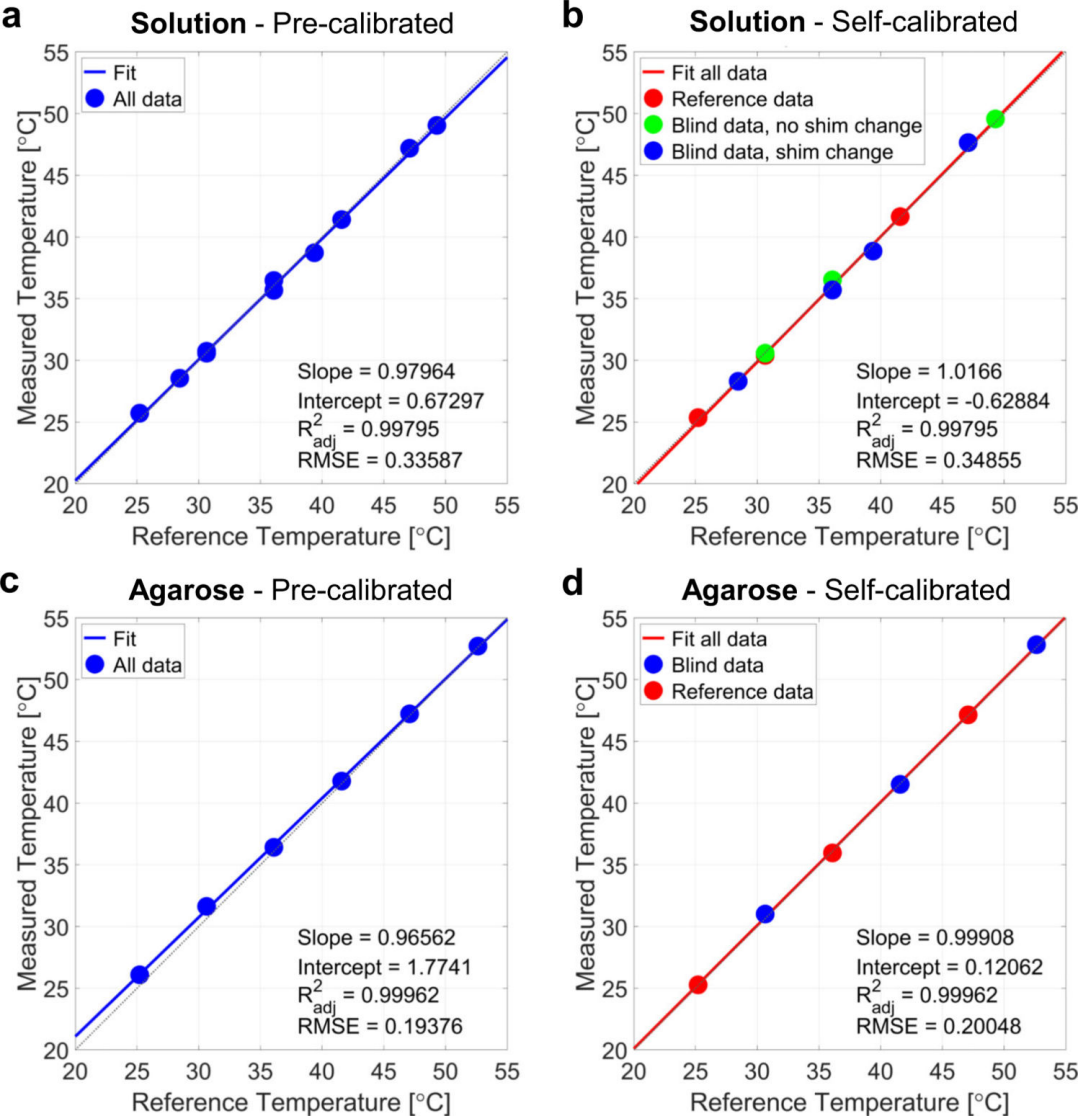
Absolute temperature measurements in solutions and agarose. **a** Absolute temperature measurements using the pre-calibrated frequency shift thermal coefficient difference Δ*α* and intercept difference Δ*σ*_0_ from the 1% NaCl solution, as a function of reference temperatures. **b** Absolute temperature measurements using the self-calibrated Δ*α* and Δ*σ*_0_ from the 1% NaCl solution, as a function of reference temperatures. The first three experiments were used to self-calibrate Δ*α* and Δ*σ*_0_ (red dots), then three temperatures were reconstructed from blind experiments at random temperatures with no shim changes (green dots), and the last four experiments included both blind temssperatures and shim changes (blue dots), all in the 1% NaCl solution. **c** Temperature measurements for the 2% agarose sample with 1% NaCl using the Δ*α* and Δ*σ*_0_ calibration from the 1% NaCl solution, as a function of reference temperatures. **d** Measured temperatures using 3 frequency measurements to self-calibrate Δ*α* and Δ*σ*_0_ (red dots) in the agarose sample, and three blind data (blue dots), as a function of reference temperatures. All temperatures are in °C.

In order to test the ability of the method to predict unknown temperatures, ten experiments were then carried out on a solution with NaCl concentration of 1% weight (similar to physiological conditions). Figure 3a shows the calculated temperatures for all the data using the Δ*α* and Δ*σ*_0_ calibration obtained with the 1% solution used in Fig. 2 and Table 1. As a next step, three peak frequency measurements at 25, 30 and 40 °C were used to self-calibrate Δ*α* and Δ*σ*_0_ for this sample, plotted by red dots in Fig. 3b. The sample was then brought to three random blind temperatures with the same shimming conditions (green dots in Fig. 3b). Then, the sample was brought to four more random blind temperatures where the magnet shims were randomly changed to alter *B*_0_ (blue dots in Fig. 3b). All calculated temperatures in Fig. 3a, b were in excellent agreement with the reference temperatures ($R_{\text{adj}}^{2}=0.998$, RMSE ~ 0:34 °C). A similar experiment was conducted in a sample with 2% agarose and 1% NaCl. Figure 3c shows the results of the calculated temperature plotted against the reference value using the pre-calibrated Δ*α* and Δ*σ*_0_ from the 1% NaCl solution used in Fig. 1c. In Fig. 3d, three frequency measurements were used to self-calibrate Δ*α* and Δ*σ*_0_ in the gel itself, and three blind temperatures were calculated. In both cases, pre-calibration in a 1% NaCl solution and self-calibration in gel led to very similar results with accurate and precise measurement of the sample temperatures ($R_{\text{adj}}^{2}=0.999$, RMSE ~ 0:20 °C).

### Ex vivo experiments

**Fig. 4:**
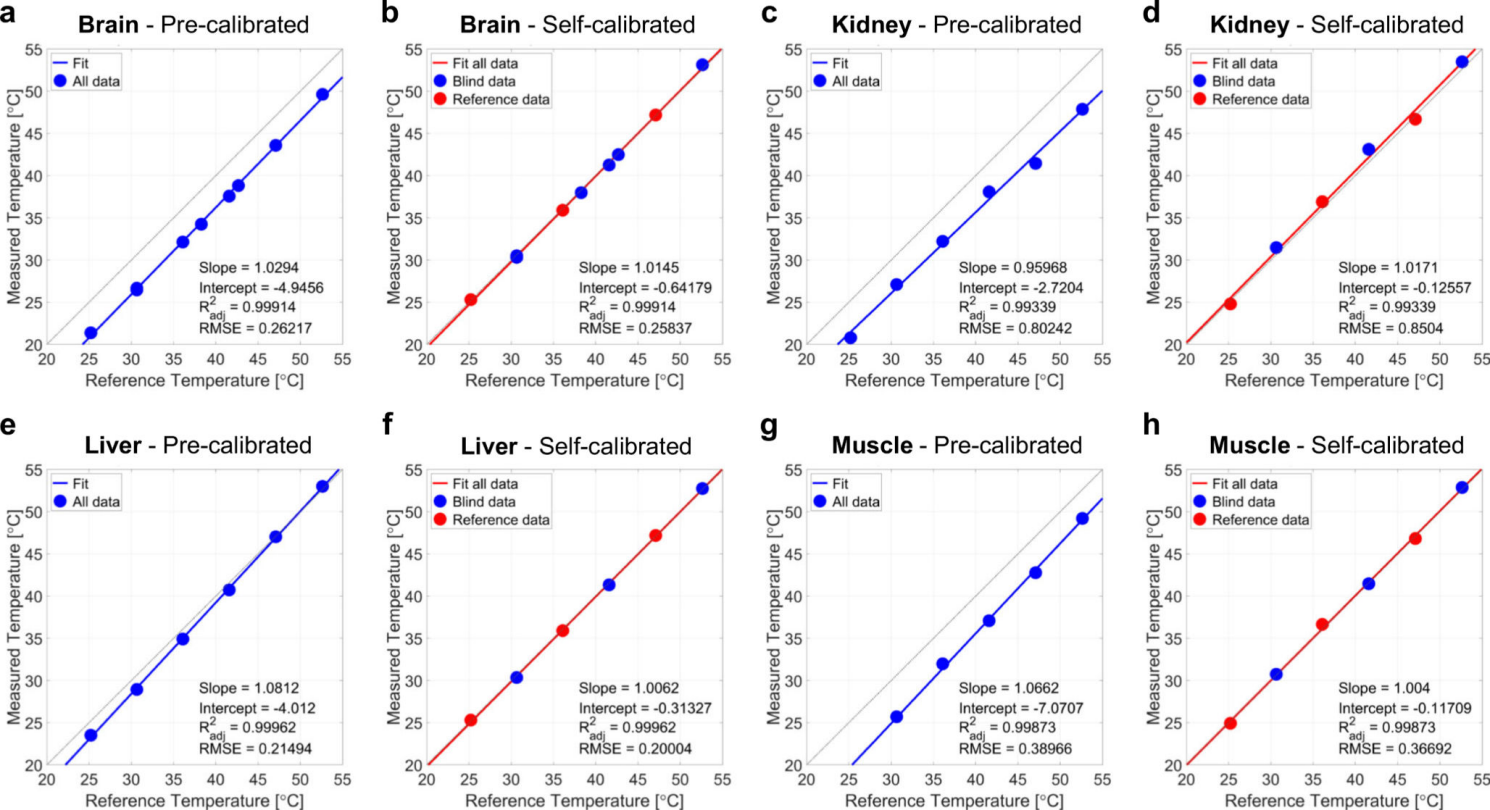
Absolute temperature measurements in ex vivo mouse tissue samples. Absolute temperature measurements in brain, kidney, liver, and muscle samples were performed using either pre-calibration of frequency shift thermal coefficient difference Δ*α* and intercept difference Δ*σ*_0_ from 0.3% NaCl solution (fitted values), or self-calibration of Δ*α* and Δ*σ*_0_ using 3 known temperatures (25, 35 and 45 °C, red dots) and blind measurements (blue dots). **a** Brain, pre-calibrated. **b** Brain, self-calibrated. **c** Kidney, pre-calibrated. **d** Kidney, self-calibrated. **e** Liver, pre-calibrated. **f** Liver, self-calibrated. **g** Muscle, pre-calibrated. **h** Muscle, self-calibrated. All temperatures are in °C.

Figure 4 shows the temperatures measured in freshly excised ex vivo mouse tissues: brain in Fig. 4a, b, kidney in Fig. 4c, d, liver in Fig. 4e, f, and muscle in Fig. 4g, h. Three peak frequency measurements at 25, 35 and 45 °C were used for self-calibrating Δ*α* and Δ*σ*_0_, and then other blind temperatures were calculated from this self-calibration. In all tissues, an excellent agreement was found for the calculated temperature when this self-calibration procedure was used, as shown in Fig. 4a, c, e, f. However, when the pre-calibration of Δ*α* and Δ*σ*_0_ was calculated from a 0.3% NaCl solution (or approximately 50 mmol/L, similar to biological tissue concentrations) from fitting of the data measured at 0.1–26% NaCl, a constant offset of 1–5 °C is detected, depending on the tissues, as shown in Fig. 4b, d, f, h. Pre-calibration of Δ*α* and Δ*σ*_0_ from the 1% NaCl and the 0.1% NaCl solution were also tested, with similar results than with 0.3% NaCl. Only in the case of liver, the pre-calibrated temperature measurement showed a good agreement with the reference temperature, as shown in Fig. 4e. The main difference in sample preparation was that the consistency of the liver sample was still homogeneous when introduced in the NMR tube, while the other tissue samples were composed of small pieces, leading to a more inhomogeneous system which increased the susceptibility effects significantly (air bubbles, fat mixture within the tissue), resulting in a constant temperature offset.

### 1D CSI experiment

**Fig. 5:**
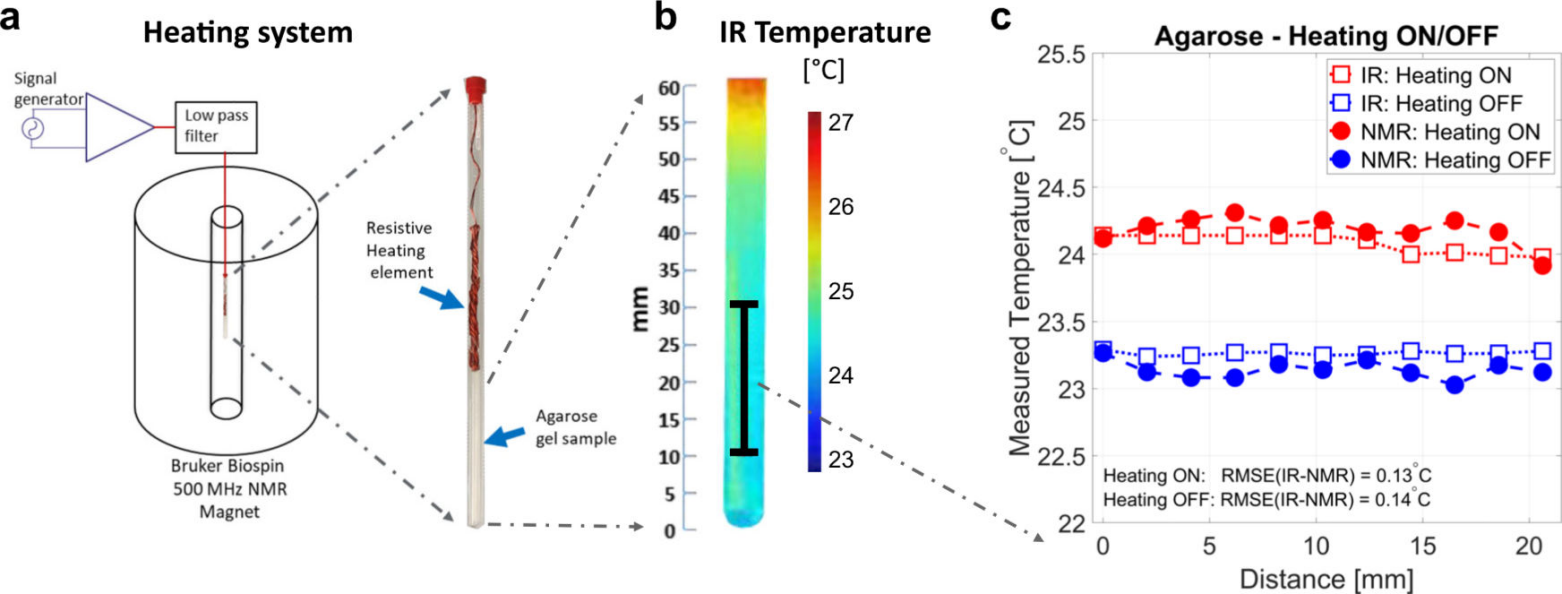
Comparison of 1D nuclear magnetic resonance (NMR) chemical shift imaging (CSI) results with infrared (IR) imaging in agarose. **a** Heating system setup. **b** Steady state temperature profile measured using IR in the distal 60 mm section of the NMR tube. The black line represents the 1D imaging volume probed using the absolute thermometry method. **c** Absolute temperature reconstructed from CSI and IR when heating was OFF and in steady state, as a function of distance (in mm) within the tube. The root mean square error (RMSE) of the difference in temperature measurements between the IR and CSI measurements was 0.13 and 0.14 °C, for the heating ON and OFF conditions, respectively.

Finally, in order to test the ability to map absolute temperature spatially, a 1D CSI measurement was carried out as shown in Fig. 5. The experiment was conducted in the gel sample with 2% agarose and 1% NaCl. The heating system setup is shown in Fig. 5a and the spatial temperature map of the sample measured with an infrared (IR) camera is shown in Fig. 5b. Figure 5c shows the measured temperatures using both IR camera (open square) and CSI data (closed circles) over 20 mm in the sample (NMR-detectable zone) before and after heating the sample. The measured temperatures using both methods are in good agreement, showing an increase of 1 °C along the entire sample after sample heating.

### Δ*α* and Δ*σ*_0_ values

The values of Δ*α* and Δ*σ*_0_ used in this paper for calculating temperatures in 1% NaCl solution, in agarose gel, and in tissue samples, are summarized in Table 1.

## Discussion

In this work, we present a new method of multinuclear absolute MR thermometry which takes advantage of the different and unique frequency shifts of the sodium and proton nuclei with temperature. The method is validated in fluid samples with different NaCl concentrations, in agarose gels, and in ex vivo fresh tissue from mice, with precise temperature control. Local magnetic field inhomogeneities are generally a challenge for thermometry methods such as the PRF. The proposed multinuclear method was shown to be less sensitive to *B*_0_ inhomogeneities upon random shim variations.

Changes in water proton frequency shifts with temperature reflect changes in the hydrogen-bonded structure of water^9,41–43^. The nature of these changes has been studied extensively, and two main models have been proposed to explain it^9,44^. In the first model, the temperature-induced frequency shift of water relates to the stretching and bending of the hydrogen bonds which are responsible for the electrical shielding effect^44^. The second model describes a change in electrical shielding due to the breaking of the hydrogen bonds. Specifically, a steady state is created between ice-like lattice water structure, where hydrogen bonds are fully formed, and a monomeric water structure where no hydrogen bonds are present. These two models, when used independently, cannot fully explain the temperature- and ionic concentration-dependent frequency shift of water. Consequently, a mixed model where hydrogen bond length stretching and bending (model 1) alongside hydrogen bond rearrangements (model 2) best explains and predicts experimental results on the temperature and ionic concentration dependency of the water frequency shift^45^. The effect of strong electrolytes (such as NaCl) causes a concentration-dependent shift in the proton resonance frequency, with some electrolytes inducing an increase in the frequency, while others, such as Na^+^, inducing a reduction in the frequency^20,46,47^. The chloride ion Cl^−^ has been shown to have a small effect on the proton frequency shift relative to that of Na^+20^. When the sodium ion is surrounded by water, a hydration shell is created, where, depending on the temperature, four to eight^48–50^ molecules of water can temporarily coordinate a single Na^+^. In such solutions, water molecules can be in an unbound state with the ion (free water outside the hydration shell), which causes minimal change to the electrostatic structure of the hydrogen bond. For a fraction of time, water molecules are in a bound state with the ion (hydration shell)^51^, causing a structural modification to the hydrogen bond, thus altering the electrical shielding of the ^1^H nucleus. The time for which water is bound to the ion is dependent on the NaCl concentration.

With respect to the frequency shift of the sodium ion, a strong correlation with the frequency shift of water was observed, suggesting that a temperature-related modification of the hydrogen bonds coexists with a modification of the electrical shielding of the sodium ion. A temperature rise increases the effective hydrogen bond length of water, increasing the negative charge distribution around the oxygen nucleus within the water molecule. This increase in negative charge distribution intensifies the ion-dipolar attraction between oxygen and sodium, consequently enhancing the electrical shielding of the sodium nucleus. As the concentration of NaCl increases, the magnitude of the frequency shift thermal coefficient *α* of sodium decreases due to the competition between the ions for the water molecules, causing a decrease in average time for which water is bound to the ion^45^. These effects form the basis for the multinuclear absolute thermometry method, enabling a sample-specific bijective mapping between the frequency difference of ^1^H and ^23^Na nuclei and temperature.

Our results demonstrate that once the proposed multinuclear thermometry method was calibrated on the aqueous solution with 1% NaCl, the frequency shift difference between the ^1^H and ^23^Na nuclei can be used to calculate the absolute temperature of the same sample under different shimming conditions with high accuracy (with an error of the order of 0.3 °C for temperatures between 25 and 50 °C). When calibration of the multinuclear thermometry method was conducted in aqueous solutions and then applied to predict the temperature in ex vivo tissue samples (brain, muscle, liver, and kidney), a constant temperature offset of 1–5 °C was observed. We believe that this offset can occur due to two main factors influencing the calibration of Δ*α* and Δ*σ*_0_: (1) the preparation of the tissue samples, and (2) the presence of multiple ions inside the tissue samples. In case (1), the tissue samples were inserted in small pieces into the 5 mm NMR tubes, thus creating relatively inhomogeneous samples with air bubbles that are artificially inducing strong local susceptibility effects which are significantly stronger than under in vivo conditions. This tissue susceptibility was not present in the aqueous solution calibration of Δ*α* and Δ*σ*_0_, and is most likely the main source of error. An exception was the liver sample that was kept uniform and homogeneous in the tube, hence a closer agreement between the pre-calibrated and the self-calibrated temperature measurements was found. In case (2), previous studies have shown that, for example, the presence of potassium ions K^+^ can cause a proton frequency shift, while other ions generally induce smaller shifts due to their small chemical shift effect or their smaller concentrations in tissues^20,47^. These ions were not present in the liquid samples, yet present in tissues at varying concentrations. The effect of these ions on the sodium resonance frequency shift is poorly understood and needs further future investigation.

Studies have shown that the volume of magnetic susceptibility changes linearly with temperature^52^, and its effect on the ^1^H resonance frequency shift is roughly an order of magnitude smaller than the electrical shielding effect^18,52,53^. As a result, calibration of the absolute thermometry method on the sample includes the sample-specific magnetic susceptibility shielding information for both sodium and proton. While susceptibility changes are accounted for in the model, measurement of temperature in voxels with very high susceptibility that alters the lineshapes of the spectra can be challenging since the reconstruction relies on detection of the proton and sodium spectra’s center frequency. This effect was observed in our CSI measurements, where voxels close to the edge of the tube and close to the metallic resistive heating apparatus had to be excluded from the reconstruction due to spectral distortion.

The absolute temperature mapping method is expected to be compatible with an implementation in vivo for potential medical applications, using either phase MRI or localized MRS at both the ^1^H and ^23^Na frequencies. Phase measurement acquisitions are more time-efficient than spectroscopic imaging as long repetition times needed to obtain high spectral resolution are not necessary, which can have an impact on the timing of clinical scanning. However, it is likely that translation of the proposed method to in vivo imaging will be challenging due to the low concentrations of sodium in vivo ranging between 15 and 150 mmol/L. These low concentrations combined with low ^23^Na NMR receptivity lead to low SNR and thus require low resolution (generally of the order of 4–6 mm isotropic) and long acquisition times (5–10 min) in MRI experiments to compensate for the loss of signal^54^. Line broadening that can be due to very short T2 relaxation in vivo (of the order of 1–15 ms), as well as potential anisotropy of the tissues, will make accurate sodium frequency estimation difficult. Moreover, MRI systems with high magnetic fields (>3 T), and multichannel dual-tuned dedicated RF coils (for brain, muscle or other organ of interest) will be necessary to increase SNR and allow concomitant proton and sodium signals detection, which, in the short term, will limit the application of this method to research centers with these capabilities. Accuracy and precision of in vivo applications will also be strongly dependent on the pre-calibration of Δ*α* and Δ*σ*_0_ for the two nuclei of interest (^1^H and ^23^Na), that should probably be performed on a wide range of ex vivo tissue samples in order to minimize uncertainties in the temperature measurements. Lastly, even within the small range of sodium concentrations present in biological tissues and fluids (15–150 mmol/L, or about 0.1–1% weight), Δ*α* and Δ*σ*_0_ can vary between tissues by about 4 × 10^5^ ppm/°C and 0.02 ppm, respectively (according to the data acquired on solutions, see Fig. 2), leading to uncertainties in accuracy of the temperature measurements of the order of 2 °C. A potential solution would be to include the quantification of the tissue sodium concentration, using internal (cerebropinal fluid, eyes) or external (gels, solutions) references, in the absolute MR thermometry procotol, and therefore correct Δ*α* and Δ*σ*_0_ for each voxel of the image before temperature calculation, using for example extrapolation from the linear fits for low sodium concentrations (<1%wt) in Fig. 2c, d. Translation and optimization of the multinuclear absolute thermometry technique to in vivo imaging, where both sodium and proton phase images can be acquired simultaneously or in an interleaved fashion^55^, will be the subject of a future investigation.

In conclusion, we present a proof-of-concept general method for measuring the absolute temperature non-invasively in samples using a multinuclear magnetic resonance approach, based on the detection of the frequency shift difference between two different nuclei (^1^H and ^23^Na is this case), and calibration of the difference of both their frequency shift thermal coefficients Δ*α* (ppm/°C) and constant intercepts Δ*σ*_0_ (ppm).

## Materials and methods

### Temperature dependence of the NMR frequency shift

The Larmor frequency *f*^*N*^ of the magnetic moment of a nucleus *N* is determined by the magnetic field *B*_nuc_ that the nucleus experiences and the gyromagnetic ratio *γ*^*N*^ of the nucleus. *B*_nuc_ is the result from a shielding constant *σ*^*N*^ altering the macroscopic magnetic field *B*_0_ according to

(1) $$f^{N}=\frac{\gamma }{2\pi }B_{\text{nuc}}=\frac{\gamma^{N}}{2\pi }\left(1-\sigma^{N}\right)B_{0}.$$

The shielding constant is expressed as

(2) $$\sigma^{N}=\sigma_{i}^{N}+\sigma_{\chi }^{N}+\sigma_{e}^{N},$$

where $\sigma_{i}^{N}$ is the intramolecular shielding constant, $\sigma_{e}^{N}$ is the intermolecular electric shielding effect, and $\sigma_{\chi }^{N}$ is the volume magnetic susceptibility shielding effect of nucleus *N*. Both $\sigma_{\chi }^{N}$ and $\sigma_{e}^{N}$ can change with temperature *T*. The precession frequency can thus be expressed as

(3) $$f^{N}(T)=\frac{\gamma^{N}}{2\pi }\left[1-\sigma_{i}^{N}-\sigma_{\chi }^{N}(T)-\sigma_{e}^{N}(T)\right]B_{0}.$$

By defining $f_{0}^{N}=\frac{\gamma^{N}}{2\pi }B_{0}$, we can calculate the frequency shift *δf*^*N*^(*T*) of a nucleus *N*, in parts-per-million (ppm), as

(4) $$\delta f^{N}(T)=\frac{f_{0}^{N}-f^{N}(T)}{f_{0}^{N}}.$$

This can be expressed as the sum of a temperature-independent component and a temperature-dependent component

(5) $$\delta f^{N}(T)=\left[\sigma_{i}^{N}\right]+\left[\sigma_{\chi }^{N}(T)+\sigma_{e}^{N}(T)\right].$$

Since the temperature dependency of $\sigma_{\chi }^{N}$ and $\sigma_{e}^{N}$ is linear with temperature^9,20^, the susceptibility and electric shielding can be written

(6a) $$\sigma_{\chi }^{N}(T)=\sigma_{\chi^{0}}^{N}+\alpha_{\chi }^{N}\cdot T,$$

(6b) $$\sigma_{e}^{N}(T)=\sigma_{e0}^{N}+\alpha_{e}^{N}\cdot T.$$

Eq. (6a) and (6b) can be combined such that the nucleus’ frequency shift is rewritten as a constant $\sigma_{0}^{N}$ (in ppm) and a frequency shift thermal coefficient *α*^*N*^ (in ppm/°C)

(7) $$\delta f^{N}(T)=\sigma_{0}^{N}+\alpha^{N}\cdot T,$$

with

(8a) $$\sigma_{0}^{N}=\sigma_{i}^{N}+\sigma_{\chi 0}^{N}+\sigma_{e0}^{N},$$

(8b) $$\alpha^{N}=\alpha_{\chi }^{N}+\alpha_{e}^{N}.$$

### Measurement of relative temperature change

The frequency shift thermal coefficient *α*^*N*^ can be calibrated for a specific nucleus (e.g., ^1^H) and a sample of interest. Since *δf*^*N*^ can vary with local *B*_0_ fluctuations (shim, motion, and field drift), and the component $\sigma_{0}^{N}$ is generally unknown and can vary due to different electronic and susceptibility shieldings, absolute temperature cannot be calculated using Eq. (7). This equation can however be used to measure relative temperature changes using nucleus *N* = ^1^H MRS or MRI (PRF method) and a calibrated value *α*^*N*^ ~ −0.01 ppm/°C^20^ in human tissues. By subtracting the frequency shifts measured at two different times (e.g., before and after heating), the effect of $\sigma_{0}^{N}$ is canceled and relative temperature changes are calculated as

(9) $$\Delta T=T_{1}-T_{2}=\frac{\delta f^{N}\left(T_{1}\right)-\delta f^{N}\left(T_{2}\right)}{\alpha^{N}}.$$

### Measurement of absolute temperature

Absolute temperature can be derived from Eq. (7) by detecting the frequency shift of two nuclei within the same sample or voxel (in case of localized MRS or MRI), where the difference between their respective frequency shift thermal coefficients *α* and constants *σ*_0_ are well-known theoretically or calibrated experimentally. Using the following definitions for two nuclei *N* ≡ *A*, *B* (which can even be of the same species, but from a different molecule or local environment)

(10a) $$\Delta f(T)=\delta f^{A}(T)-\delta f^{B}(T),$$

(10b) $$\Delta \sigma_{0}=\sigma_{0}^{A}-\sigma_{o}^{B},$$

(10c) $$\Delta \alpha =\alpha^{A}-\alpha^{B}\neq 0,$$

the frequency shift difference between the two nuclei can thus be written

(11) $$\Delta f(T)=\Delta \sigma_{0}+\Delta \alpha \cdot T.$$

Upon calibration of Δ*σ*_0_ and Δ*α* for the two nuclei and samples of interest (fluid, tissue), absolute temperature of the sample can be calculated as follows

(12) $$T=\frac{\Delta f(T)-\Delta \sigma_{0}}{\Delta \alpha }.$$

In the present study, we propose to measure the absolute temperature using two different nuclei, ^1^H and ^23^Na, which both exhibit a unique frequency dependency with temperature. The two nuclei are conjointly present in a hydrated sample (and thus experience the same local *B*_0_ variations, as well as similar electronic and susceptibility environments), as sodium ions Na^+^ are mostly present in hydrated state in the water compartment of the body or a sample of interest.

We measured the NMR frequency shifts of ^1^H (hydrogen from water) and ^23^Na (from ion Na^+^) nuclei at different temperatures in solutions with different NaCl concentrations, as well as in agarose gel and in ex vivo mouse tissue samples, in order to measure their respective linear dependence with temperature and calibrate their respective Δ*α* and Δ*σ*_0_. These two latter values were then used to calculate the absolute temperature of the samples in blind experiments, where the temperature of the sample was known from the spectrometer sensor, but not used for the calibration of Δ*α* and Δ*σ*_0_.

### NMR experiments

Experiments were carried out on an 11.7 T NMR Bruker Avance I spectrometer (Bruker BioSpin) operating at 500.19 MHz for ^1^H, and 132.3 MHz for ^23^Na, using a 5 mm double resonance broadband probe. The test tubes with different samples under investigation (aqueous solutions with different NaCl concentrations, agarose gel, ex vivo tissues) were placed inside the spectrometer where the sample temperature could be controlled using gas flow and a temperature sensor providing a precise, stable and reliable temperature regulation. After each desired temperature was reached, a standard free induction decay (FID) pulse sequence was used with a 90° pulse. The duration of the pulse is 11 and 9 μs for ^1^H and ^23^Na, respectively, and 8 averages were used with TR = 15 s for ^1^H, and 0.5 s for ^23^Na, dwell time dw = 100 μs, spectral width sw = 5 kHz, 16,384 data points per spectrum. Complex FIDs were acquired in digital quadrature detection (DQD) mode, a simultaneous acquisition mode in Bruker systems resulting in $sw=\frac{1}{2\mathrm{dw}}$. All experiments were performed with the following exact spectrometer reference frequencies: $f_{0}^{\text{H}}=500.2031765\;\text{MHz}$, $f_{0}^{\text{Na}}=132.3120951\;\text{MHz}$ (fixed ratio $f_{0}^{\text{H}}/f_{0}^{\text{Na}}=3.7804796$).

### Sample preparation

Solution samples with 11 different NaCl concentrations (*C* = 0.1, 1, 2, 5, 8, 11, 14, 17, 20, 23, 26% weight) were prepared by mixing x mg of NaCl in (*y* – *x*) mg of deionized water in a beaker (with *x* = 0.1 mg and *y* = 100 mg for the sample *C* = 0.1% weight, and with *x* = 0.1, 0.2, 0.5, 0.8, 1.1, 1.4, 1.7, 2.0, 2.3, 2.6 mg and *y* = 10 mg for the other samples), and transferred to 5 mm NMR tubes (sample volume = 0.5 mL). All mass measurements were performed on a Mettler Toledo ME204E balance with a resolution of 0.1 mg. The solution at 26% weight correspond to NaCl saturation in water. Corresponding NaCl concentrations in mol/L and uncertainties on the measurements can be calculated as described in Supplementary Note 2. Results of the calculated NaCl concentrations in mol/L with uncertainties are presented in Supplementary Table 2. A gel was prepared by mixing 2% w/v of agarose with 1% w/v NaCl in deionized water. The gel mixture was incrementally heated in a microwave to fully dissolve the agarose. The solution was poured into a 5 mm NMR tube forming a uniform, homogeneous gel upon cooling.

### Tissue samples

Four tissues samples (brain, kidney, liver, and muscle) were obtained from two female mice whose weights were 22.2 and 25 g.

### Data processing

The frequency shifts of the ^1^H and ^23^Na signals were detected at each temperature by tracking the position of the maximum of peak of their NMR spectrum: (1) The maximum of each magnitude spectrum was detected and 256 data points around this maximum were selected (128 points on each side); (2) the 257 data points (including the maximum point) were then fitted by a Lorentzian function; (3) the maximum of the Lorentzian fit was detected and its corresponding frequency was selected as the frequency shift of interest for this particular spectrum. Although this maximum-of-fit detection method was not necessary for solution and gel samples, it proved to slightly improve the robustness of the frequency shift detection in tissue samples, particularly when SNR was low or when the peaks where distorted due to local susceptibility inhomogeneities. Examples of fitting results for both ^1^H and ^23^Na spectra from the muscle sample at different temperatures are shown in Supplementary Fig. 13. For comparison, we also included three examples of fits of the whole spectra in the muscle sample using a bi-Lorentzian function, at three different temperatures in Supplementary Fig. 14. Due to variations in spectra shapes caused by both global and local magnetic field inhomogeneities in our biological samples, we found that the (256+1)-point fitting method used to track the frequency shift as described above was more robust compared to whole spectra fitting. All data processing was performed in Matlab (The MathWorks Inc., Natick, MA, USA).

### Measurements of *α* and *σ*_0_ for ^1^H and ^23^Na in solutions

The frequency shift thermal coefficient *α* (ppm/°C) and constant intercept *σ*_0_ (ppm) were measured in 11 solutions with different NaCl concentrations (*C* = 0.1, 1, 2, 5, 8, 11, 14, 17, 20, 23, 26% weight), by fitting the frequency shift *f* (ppm) of the maximum of the NMR peak versus 6 temperatures (*T* = 25, 30, 35, 40, 45, 50 °C), for both the ^1^H and ^23^Na nuclei

(13) $$f=\alpha T+\sigma_{0}.$$

Data and fits are shown in Supplementary Figs. 1–11, and *α* and *σ*_0_ values are summarized in Supplementary Table 3. Fittings of *α*, Δ*α*, Δ*σ*_0_ versus NaCl concentrations in weight % (*C*_%wt_) were also performed as described in Supplementary Note 3, and results are shown in Supplementary Table 4.

### Effect of pH

In order to study the effect of pH on the multinuclear MR temperature measurements, solutions with different pH values were tested for Δ*α* and Δ*σ*_0_ calibration. The solutions of different pH values were prepared by adding a small amount of acid HCl or base KOH solutions to the water solution sample with 1% weight NaCl, to adjust to the desired pH value. The pH was measured with a Fisher Scientific^PM^ accumet^PM^ AB150 pH Benchtop Meter and calibrated with three standard buffers with pH values 4.01, 7, and 10.01. The reported pH values were measured before acquiring the NMR data. The pH range was from 4.9 to 9.07. The results are summarized in Supplementary Fig. 12 and demonstrate that pH has negligible influence on the Δ*α* and Δ*σ*_0_ values.

### Heating system and 1D CSI procedure

An in-house built alternating-current resistive heating setup was constructed to create an NMR-compatible heating setup that does not interfere with the multinuclear NMR acquisition^56^. A signal generator (B071HJ31WN, KKmoon, China), operating at 100 kHz was connected to a 130W class D amplifier (TPA3250D2EVM, Texas instruments Inc., USA). The output of the amplifier was connected an in-house built low pass filter with a cutoff frequency of 10 MHz to mitigate RF waves being picked up and transmitted in close proximity to the RF coil in the NMR spectrometer. The output of the low pass filter was connected to a resistive wire insert made of wound AWG 32G enameled copper wire (ECW32AWG1LB, Bntechgo Inc., USA) placed inside the 5 mm NMR test tube filled with 2% agarose and 1% NaCl in water. A baseline proton 1D CSI acquisition was conducted with the following imaging parameters: 16 steps in the z-encoding, 1 average, and a repetition time of 15 s, giving a total experimental time of 5 min. A sodium 1D CSI acquisition over the same field of view was then acquired with the following parameters: 16 steps in the z-encoding, 32 averages, and a repetition time of 0.3 s, with a total experimental time of 5 min. The 1D CSI pulse sequence consisted of a 90° pulse followed by a pulse gradient which encodes the spatial position in *z*-direction. After the baseline proton and sodium acquisitions were conducted, a 1V peak-to-peak sinusoidal waveform was used to drive the amplifier. The waveform at 100 kHz was used in order to not interfere with the RF, gradient or *B*_0_ field. Sample temperature was monitored in real time with the internal temperature probe of the Bruker 500 MHz spectrometer to ensure that heating of the sample was in a steady state. After twenty minutes, a steady state of the temperature was attained, and CSI acquisitions were acquired at proton and sodium frequencies. Sodium and proton spectra were then used to reconstruct the absolute temperature. The absolute temperature was plotted and compared with IR temperature measurements acquired at steady state temperature using a FLAIR IR camera (E75, FLIR Systems Inc., USA).

## Supplementary Material

SIlleta SuplInfo

## Figures and Tables

**Table 1:** Δ*α* and Δ*σ*_0_ for different samples. Frequency shift thermal coefficient difference Δ*α* = *α*(^1^H)−*α*(^23^Na) (in ppm/°C), and intercept difference Δ*σ*_0_ = *σ*_0_(^1^H)−*σ*_0_(^23^Na) (in ppm), were measured in different samples, either using self-calibration from the sample itself or pre-calibration from the solutions with 0.1–26% weight of NaCl.

Samples	Calibration	Δα (ppm/°C)	Δσ_0_ (ppm)
Solution 1%	Solutions 0.1–26%	0.010690	0.056744
Solution 1%	Self-calibration	0.010301	0.070416
Solution 0.3%	Solutions 0.1–26% (fit)	0.010720	0.075141
Agarose	Self-calibration	0.010321	0.075775
Brain	Self-calibration	0.011060	0.023918
Muscle	Self-calibration	0.011887	−0.01556
Kidney	Self-calibration	0.011206	0.029343
Liver	Self-calibration	0.011500	0.034852

## References

[1] Rieke V & Butts Pauly K MR thermometry. J. Magn. Reson. Imaging 27, 276–390 (2008). 18219673 10.1002/jmri.21265 PMC2780364

[2] Bloembergen N, Purcell EM & Pound RV Relaxation effects in nuclear magnetic resonance absorption. Phys. Rev. 73, 679 (1948).

[3] Simpson J & Carr H Diffusion and nuclear spin relaxation in water. Phys. Rev. 111, 1201 (1958).

[4] Nelson T & Tung S Temperature dependence of proton relaxation times in vitro. Magn. Res. Imaging 5, 189–199 (1987). 10.1016/0730-725x(87)90020-8 3041151

[5] Hall AS, Prior MV, Hand JW, Young IR & Dickinson RJ Observation by MR imaging of in vivo temperature changes induced by radio frequency hyperthermia. J. Comput. Assist Tomogr. 14, 430–436 (1990). 2335613 10.1097/00004728-199005000-00021

[6] Dickinson R, Hall A, Hind A & Young I Measurement of changes in tissue temperature using MR imaging. J Comput. Assist Tomogr. 10, 468–472 (1986). 3700752

[7] Bottomley PA, Foster TH, Argersinger RE & Pfeifer LM A review of normal tissue hydrogen NMR relaxation times and relaxation mechanisms from 1–100 MHz: dependence on tissue type, NMR frequency, temperature, species, excision, and age. Med. Phys. 11, 425–448 (1984). 6482839 10.1118/1.595535

[8] Delannoy J, Chen C-N, Turner R, Levin R & Le Bihan D Noninvasive temperature imaging using diffusion MRI. Magn. Res. Med. 19, 333–339 (1991). 10.1002/mrm.1910190224 1881323

[9] Hindman J Proton resonance shift of water in the gas and liquid states. J. Chem. Phys. 44, 4582–4592 (1966).

[10] Ishihara Y A precise and fast temperature mapping using water proton chemical shift. Magn. Res. Med. 34, 814–823 (1995). 10.1002/mrm.1910340606 8598808

[11] Poorter JD Noninvasive MRI thermometry with the proton resonance frequency (PRF) method: in vivo results in human muscle. Magn. Res. Med. 33, 74–81 (1995). 10.1002/mrm.1910330111 7891538

[12] Holbrook AB, Santos JM, Kaye E, Rieke V & Pauly KB Real-time MR thermometry for monitoring HIFU ablations of the liver. Magn. Res. Med. 63, 365–373 (2010). 10.1002/mrm.22206 PMC3212435 19950255

[13] Roujol S, de Senneville BD, Hey S, Moonen C & Ries M Robust adaptive extended Kalman filtering for real time MR-thermometry guided HIFU interventions. IEEE Trans. Med. Imaging 31, 533–542 (2012). 21997254 10.1109/TMI.2011.2171772

[14] Kickhefel A, Roland J, Weiss C & Schick F Accuracy of real-time MR temperature mapping in the brain: a comparison of fast sequences. Phys. Med. 26, 192–201 (2010). 20096617 10.1016/j.ejmp.2009.11.006

[15] Delannoy J, LeBihan D, Hoult D & Levin R Hyperthermia system combined with a magnetic resonance imaging unit. Med. Phys. 17, 855–860 (1990). 2233572 10.1118/1.596477

[16] van den Bosch M MRI-guided radiofrequency ablation of breast cancer: preliminary clinical experience. J. Magn. Res. Imaging 27, 204–208 (2008). 10.1002/jmri.21190 18050333

[17] Alon L, Cho GY, Yang X, Sodickson DK & Deniz CM A method for safety testing of radiofrequency/microwave-emitting devices using MRI. Magn. Res. Med. 74, 1397–1405 (2015). 10.1002/mrm.25521 PMC4442746 25424724

[18] Poorter JD Noninvasive MRI thermometry with the proton resonance frequency method: study of susceptibility effects. Magn. Res. Med. 34, 359–367 (1995). 10.1002/mrm.1910340313 7500875

[19] Young IR An evaluation of the effects of susceptibility changes on the water chemical shift method of temperature measurement in human peripheral muscle. Magn. Res. Med. 36, 366–374 (1996). 10.1002/mrm.1910360307 8875406

[20] Hindman J Nuclear magnetic resonance effects in aqueous solutions of 1–1 electrolytes. J. Chem. Phys. 36, 1000–1016 (1962).

[21] Rieke V Referenceless MR thermometry for monitoring thermal ablation in the prostate. IEEE Trans. Med. Imag. 26, 813–821 (2007). 10.1109/TMI.2007.892647 PMC2780365 17679332

[22] Vigen KK, Daniel BL, Pauly JM & Butts K Triggered, navigated, multi-baseline method for proton resonance frequency temperature mapping with respiratory motion. Magn. Res. Med. 50, 1003–1010 (2003). 10.1002/mrm.10608 14587011

[23] Peters RD Magnetic Resonance Thermometry For Image-guided Thermal Therapy. Ph.D. thesis. (University of Toronto, 2000).

[24] van Rhoon GC Is CEM43 still a relevant thermal dose parameter for hyperthermia treatment monitoring? Int. J. Hypertherm 32, 50–62 (2016). 10.3109/02656736.2015.1114153 26758036

[25] van Rhoon GC CEM43° c thermal dose thresholds: a potential guide for magnetic resonance radiofrequency exposure levels? Eur. Rad. 23, 2215–2227 (2013). 10.1007/s00330-013-2825-y PMC3799975 23553588

[26] Leavitt V, Kangarlu A, Liu F, Riley C & Sumowski J Elevated brain temperature is associated with worse fatigue in relapsing remitting multiple sclerosis patients. Neurology 86 2 172 (2016).

[27] Wang H Brain temperature and its fundamental properties: a review for clinical neuroscientists. Front. Neurosci. 8, 307 (2014). 25339859 10.3389/fnins.2014.00307 PMC4189373

[28] Tang X, Ding H, e Yuan Y & Wang Q Morphological measurement of localized temperature increase amplitudes in breast infrared thermograms and its clinical application. Biomed. Signal Process. Control 3, 312–318 (2008).

[29] Zaretsky DV, Romanovsky AA, Zaretskaia MV & Molkov YI Tissue oxidative metabolism can increase the difference between local temperature and arterial blood temperature by up to 1.3oc: implications for brain, brown adipose tissue, and muscle physiology. Temperature 5, 22–35 (2018). 10.1080/23328940.2018.1437311 PMC5902193 29687042

[30] Raiford DS, Fisk CL & Becker ED Calibration of methanol and ethylene glycol nuclear magnetic resonance thermometers. Anal. Chem. 51, 2050–2051 (1979).

[31] Van Geet AL Calibration of the methanol and glycol nuclear magnetic resonance thermometers with a static thermistor probe. Anal. Chem. 40, 2227–2229 (1968).

[32] Raiford DS, Fisk CL & Becker ED Calibration of methanol and ethylene glycol nuclear magnetic resonance thermometers. Anal. Chem. 51, 2050–2051 (1979).

[33] Rigotti DJ Longitudinal whole-brain n-acetylaspartate concentration in healthy adults. Am. J. Neurorad. 32, 1011–1015 (2011). 10.3174/ajnr.A2452 PMC3129626 21511862

[34] Dehkharghani S Proton resonance frequency chemical shift thermometry: experimental design and validation toward high-resolution noninvasive temperature monitoring and in vivo experience in a nonhuman primate model of acute ischemic stroke. Am. J. Neurorad. 36, 1128–1135 (2015). 10.3174/ajnr.A4241 PMC4894329 25655874

[35] Alon L, Deniz CM, Brown R, Sodickson DK & Zhu Y Method for in situ characterization of radiofrequency heating in parallel transmit MRI. Magn. Res. Med 69, 1457–1465 (2013). 10.1002/mrm.24374 PMC3449021 22714806

[36] Oh S, Ryu Y-C, Carluccio G, Sica CT & Collins CM Measurement of SAR-induced temperature increase in a phantom and in vivo with comparison to numerical simulation. Magn. Res. Med. 71, 1923–1931 (2014). 10.1002/mrm.24820 PMC3842374 23804188

[37] McDannold N Temperature mapping considerations in the breast with line scan echo planar spectroscopic imaging. Magn. Reson. Med. 58, 1117–1123 (2007). 18046702 10.1002/mrm.21322

[38] Baron P Influence of water and fat heterogeneity on fat-referenced MR thermometry. Magn. Reson. Med. 75, 1187–1197 (2016). 25940426 10.1002/mrm.25727

[39] Zhang L Accurate MR thermometry by hyperpolarized 129Xe. Magn. Reson. Med. 78, 1070–1079 (2017). 27759913 10.1002/mrm.26506 PMC5757877

[40] Schilling F MRI thermometry based on encapsulated hyperpolarized xenon. ChemPhysChem 11, 3529–3533 (2010). 20821795 10.1002/cphc.201000507

[41] Schneider WG, Bernstein H & Pople J Proton magnetic resonance chemical shift of free (gaseous) and associated (liquid) hydride molecules. J. Chem. Phys. 28, 601–607 (1958).

[42] Muller N Concerning structural models for water and chemical-shift data. J. Chem. Phys. 43, 2555–2556 (1965).

[43] Rüterjans HH & Scheraga HA Chemical-shift data for water and aqueous solutions. J. Chem. Phys. 45, 3296–3298 (1966).

[44] Pople JA Molecular association in liquids II. A theory of the structure of water. Proc. R. Soc Lon. Ser. A Math. Phys. Sci. 205, 163–178 (1951).

[45] Hindman J Proton magnetic resonance studies of water structure In (eds. Baer W, Perkins A and EL G) Developments in Applied Spectroscopy, vol. 6, 251–263 (Springer, Boston, MA, USA, 1968).

[46] Wertz JE & Jardetzky O Nuclear spin resonance of aqueous sodium ion. J. Chem. Phys. 25, 357–358 (1956).

[47] Shoolery JN & Alder BJ Nuclear magnetic resonance in concentrated aqueous electrolytes. J. Chem. Phys. 23, 805–811 (1955).

[48] Ohtaki H & Fukushima N A structural study of saturated aqueous solutions of some alkali halides by x-ray diffraction. J. Sol. Chem. 21, 23–38 (1992).

[49] Mancinelli R, Botti A, Bruni F, Ricci M & Soper A Hydration of sodium, potassium, and chloride ions in solution and the concept of structure maker/breaker. J. Phys. Chem. B 111, 13570–13577 (2007). 17988114 10.1021/jp075913v

[50] Chizhik V, Mikhailov V & Su PC NMR relaxation data on the microstructure of aqueous solutions of alkali-metal salts and hydroxides. Theor. Exp. Chem. 22, 480–483 (1987).

[51] Malinowski ER, Knapp PS & Feuer B NMR studies of aqueous electrolyte solutions. I. Hydration number of NaCl determined from temperature effects on proton shift. J. Chem. Phys. 45, 4274–4279 (1966).

[52] Peters RD, Hinks RS & Henkelman RM Heat-source orientation and geometry dependence in proton-resonance frequency shift magnetic resonance thermometry. Magn. Res. Med. 41, 909–918 (1999). 10.1002/(sici)1522-2594(199905)41:5<909::aid-mrm9>3.0.co;2-n 10332873

[53] Odéen H & Parker DL Magnetic resonance thermometry and its biological applications—physical principles and practical considerations. Prog. Nuclear Magn. Reson. Spectrosc. 110, 34–61 (2019). 10.1016/j.pnmrs.2019.01.003 PMC6662927 30803693

[54] Madelin G & Regatte RR Biomedical applications of sodium MRI in vivo. J. Magn. Reson. Imaging 38, 511–529 (2013). 23722972 10.1002/jmri.24168 PMC3759542

[55] Lee S, Hilal S & Cho Z A multinuclear magnetic resonance imaging technique-simultaneous proton and sodium imaging. Magn. Reson. Imaging 4, 343–350 (1986). 2823046 10.1016/0730-725x(86)91044-1

[56] Gilchrist S An MRI-compatible high frequency AC resistive heating system for homeothermic maintenance in small animals. PLoS ONE 11, e0164920 (2016). 27806062 10.1371/journal.pone.0164920 PMC5091850

