# Supplementary material for: Multinuclear absolute magnetic resonance thermometry
Source: Commun Phys. Author manuscript; Available in PMC 2020 Oct 15. (PMC7561043; doi:10.1038/s42005-019-0252-3)
Supplement: SIlleta SuplInfo [file NIHMS1612358-supplement-SIlleta_SuplInfo.pdf]

Supplementary Note 1: Spectrometer temperature calibration

The sample temperature was controlled with a variable temperature system, which is part of the Bruker spectrometer. The gas flow streams through a pipe along the sample tube and leaves the probe head at the top. A temperature sensor measures the temperature and gives the value to a control unit that regulates the heater power to keep the temperature constant. Since the temperature sensor is not inside the NMR tube, a calibration must be done in a sample with a known temperature-dependence behavior. Calibration data was previously acquired on this spectrometer on methanol, where the chemical shift difference between the peaks correlates to the real temperature (1). The range of temperatures was from -50°C to 67°C. The real temperatures calculated from the frequency shifts of the peaks are compared to the temperatures obtained from the control unit in the spectrometer, and the following fitting parameters were obtained between the real temperature T_{real} ($^{\circ}C$) in the sample and the temperature measured by the spectrometer sensor T_{spec} ($^{\circ}C$):

$$T_{real} = a \cdot T_{spec}^2 + b \cdot T_{spec} + c, \quad [1]$$

with a = 5.944181×10^{-4}, b = 1.052388, and c = -1.470807. We used the same correction for all of our experiments. See Supplementary Table 1 for the resulting corrected (real) temperatures used in our experiments.

Supplementary Note 2: NaCl concentration calibration

The conversion of NaCl concentration C from % weight (%wt) unit to mol/L unit was calculated using the two following equations:

$$\rho = a \cdot C_{\%wt}^2 + b \cdot C_{\%wt} + c, \quad [2]$$

with the density of water ρ in kg/L or g/mL, a = 1.682×10^{-5}, b = 0.007079 and c = 0.9984, calculated using the data from the CRC Handbook of Chemistry and Physics (86th ed), p. 8-71 (2), on the properties of water-NaCl mixtures (density of water at different NaCl concentrations in %wt); and

$$C_{mol/L} = \frac{C_{\%wt}}{100} \times \frac{\rho}{M} \times 1000, \quad [3]$$

with M = 58.44 g/mol the molar mass of NaCl. Results are presented in Supplementary Table 2.

Uncertainty calculation. All mass measurements were performed on a Mettler Toledo ME204E balance with a resolution of 0.1 mg according to the following method in order to decrease imprecision: (1) the balance was calibrated to zero before each measurement, (2) NaCl was added in very small amounts on the balance until the value measured increased by a step of 0.1 mg, and this until reaching the expected value for each sample. This method can thus be estimated to reasonably results in a measurement uncertainty of ±0.5 mg (or 0.1 mg uncertainty around the expected value). According to Equations 2 and 3, and standard error propagation (3),

$$C_{mol/L} = \frac{10}{M} \times \left(a \cdot C_{\%wt}^3 + b \cdot C_{\%wt}^2 + c \cdot C_{\%wt} \right), \quad [4]$$

and the uncertainty $\sigma_{C_{mol/L}}$ on $C_{mol/L}$ is:

$$\sigma_{C_{mol/L}} = \frac{\partial C_{mol/L}}{\partial C_{\%wt}} \times \sigma_{C_{\%wt}} = \frac{10}{M} \times \left(3a \cdot C_{\%wt}^2 + 2b \cdot C_{\%wt} + c \cdot \right) \times \sigma_{C_{\%wt}}, \quad [5]$$

with $\sigma_{C_{\%wt}} = 0.05\%$ for $C_{\%wt} = 0.1\%$, and 0.5% for all other samples, according to the mass measurements for preparing the samples and balance precision. Results are presented in Supplementary Table 2.

Supplementary Note 3: Fitting of α, $\Delta\alpha$, $\Delta\sigma_0$ vs. NaCl concentrations

The values of α (ppm/°C), and $\Delta\alpha$ (ppm/°C), information document. measured at different NaCl concentrations $C_{\%wt}$ were fitted using the equations below.
For α of ^{1}H and ^{23}Na, and for $\Delta\alpha$:

$$\alpha = a \cdot C_{\%wt} + b. \quad [6a]$$

$$\Delta\alpha = a \cdot C_{\%wt} + b. \quad [6b]$$

For $\Delta\sigma_0$:

$$\Delta\sigma_0 = a \cdot C_{\%wt}^2 + b \cdot C_{\%wt} + c. \quad [7]$$

The fitting parameters are given in Supplementary Table 4.

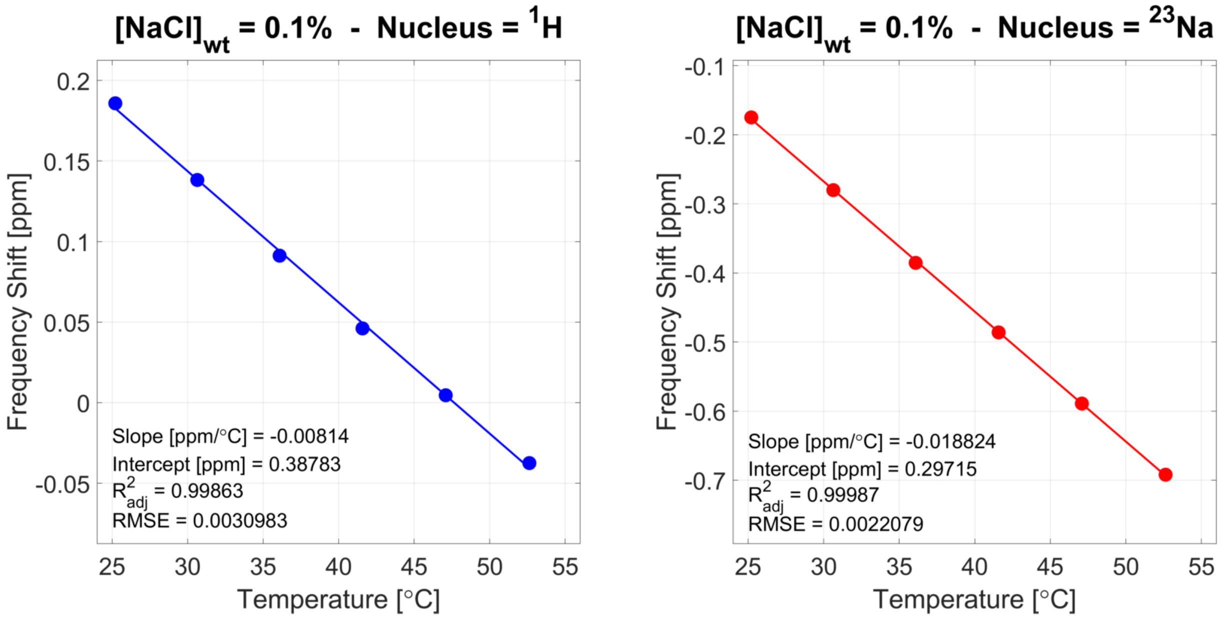

Supplementary Figure 1. Proton (^{1}H) and sodium (^{23}Na) spectra frequency shifts at different temperatures for solution with [NaCl] = 0.1% weight.

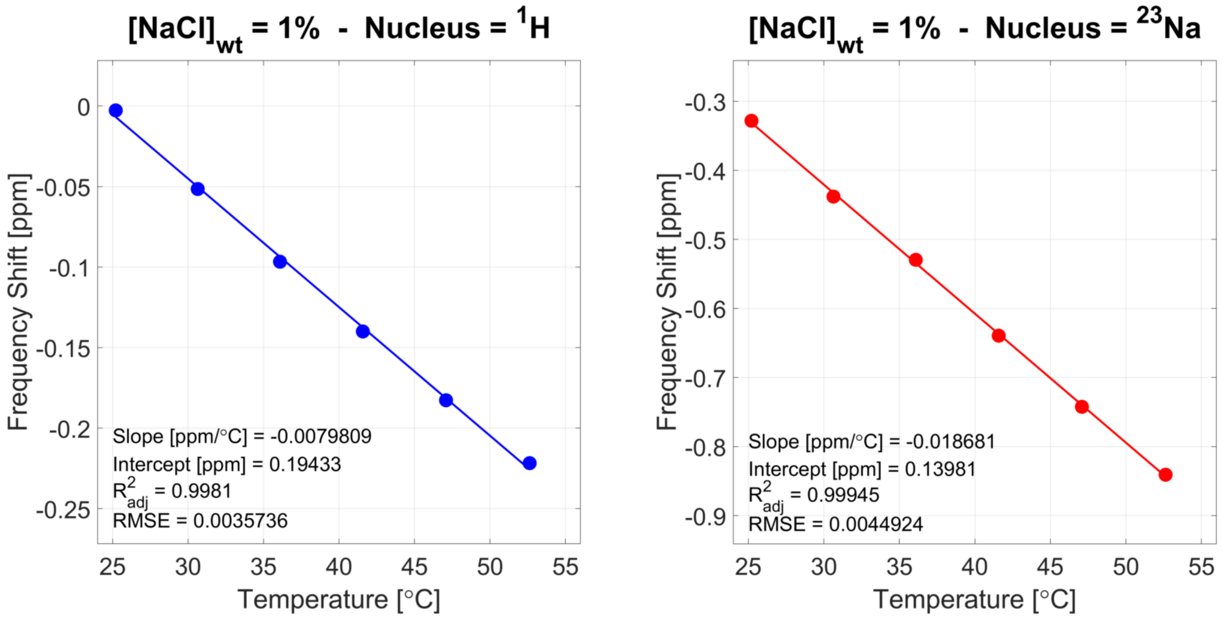

Supplementary Figure 2. Proton (^{1}H) and sodium (^{23}Na) spectra frequency shifts at different temperatures for solution with [NaCl] = 1% weight.

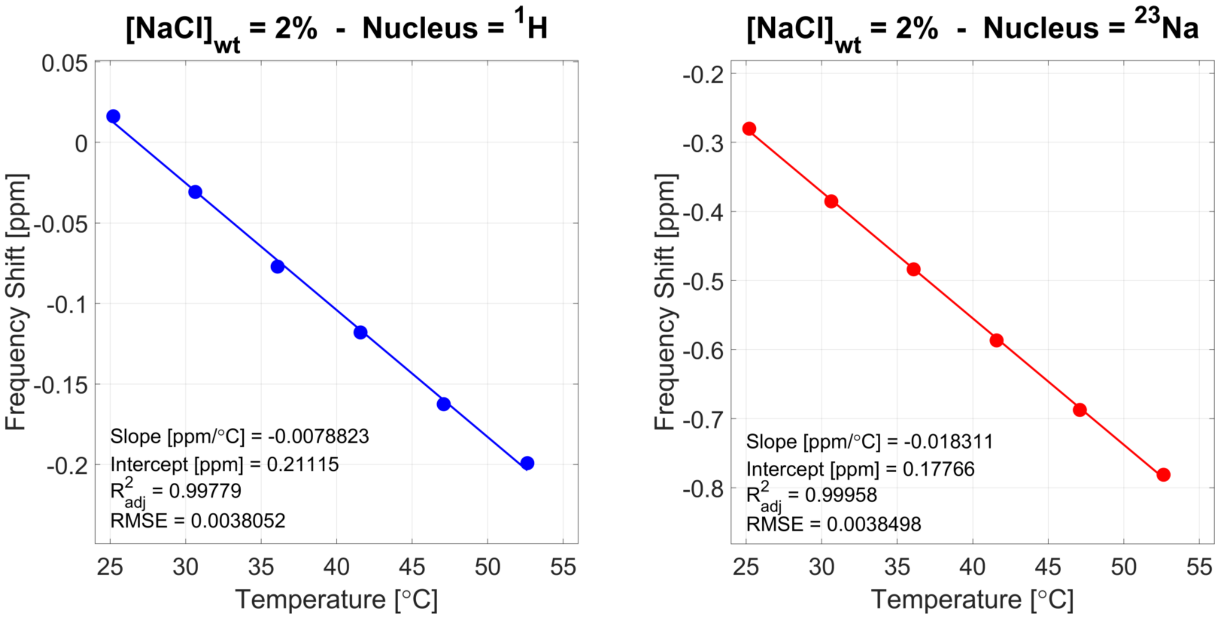

Supplementary Figure 3. Proton (^1H) and sodium (^{23}Na) spectra frequency shifts at different temperatures for solution with [NaCl] = 2% weight.

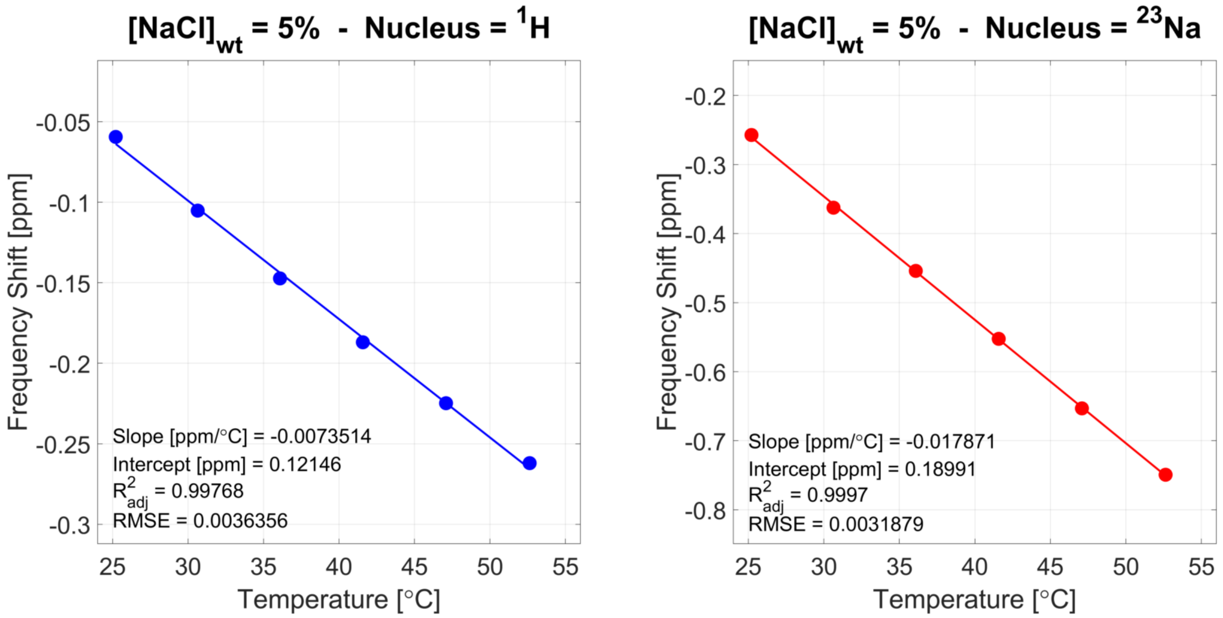

Supplementary Figure 4. Proton (^1H) and sodium (^{23}Na) spectra frequency shifts at different temperatures for solution with [NaCl] = 5% weight.

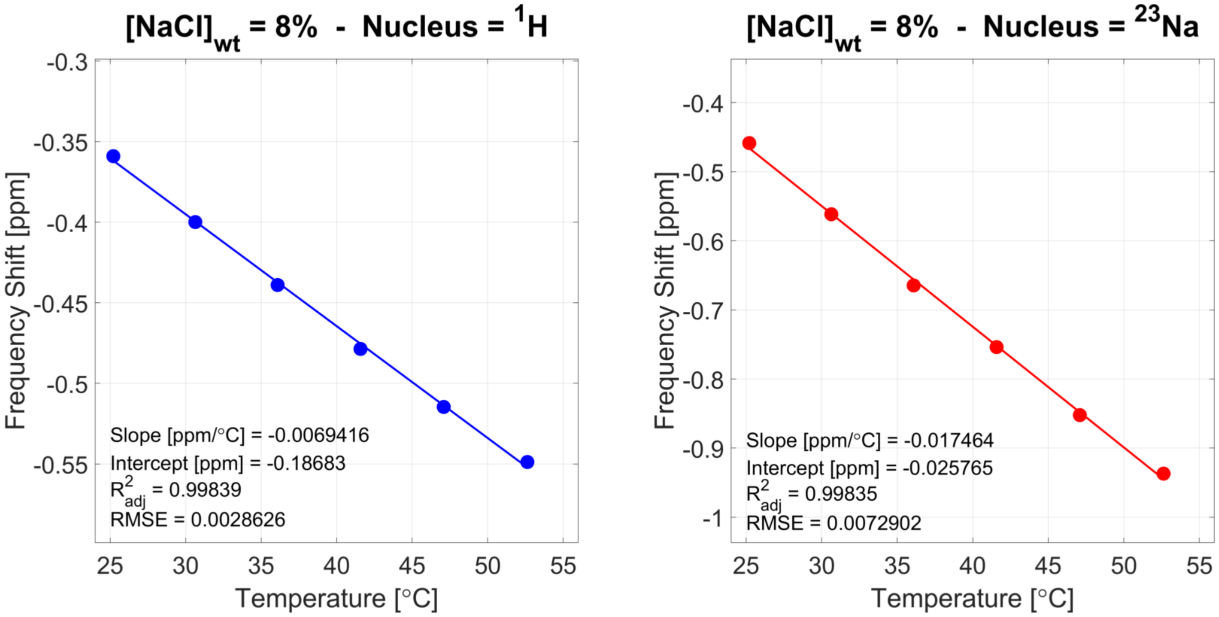

Supplementary Figure 5. Proton (^{1}H) and sodium (^{23}Na) spectra frequency shifts at different temperatures for solution with [NaCl] = 8% weight.

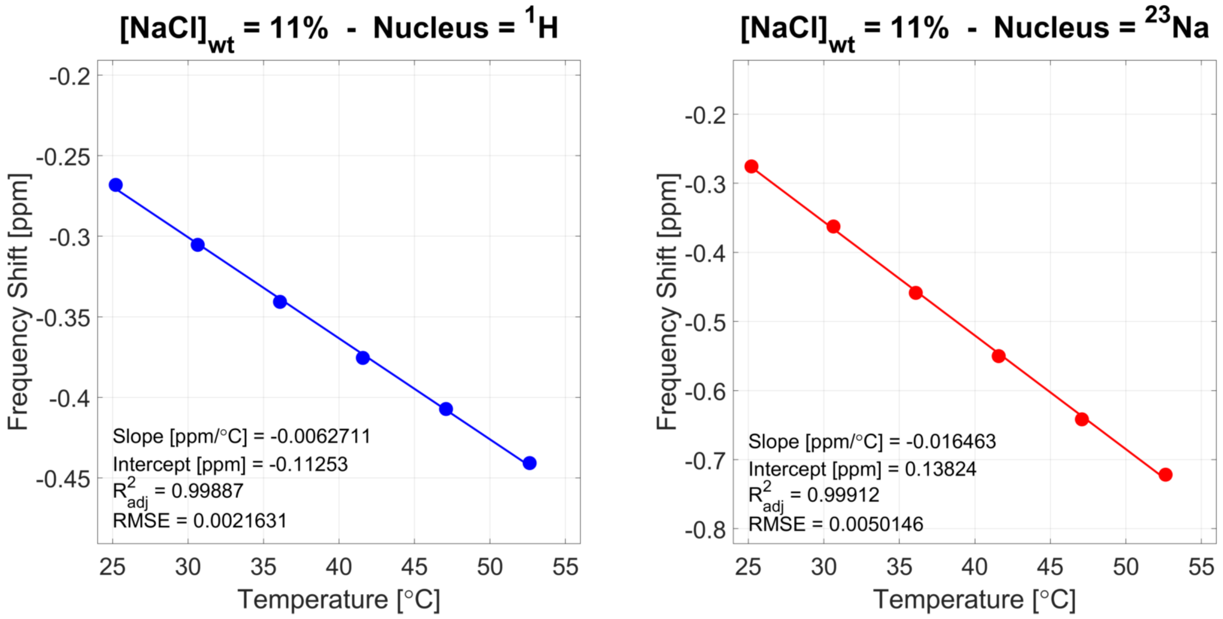

Supplementary Figure 6. Proton (^{1}H) and sodium (^{23}Na) spectra frequency shifts at different temperatures for solution with [NaCl] = 11% weight.

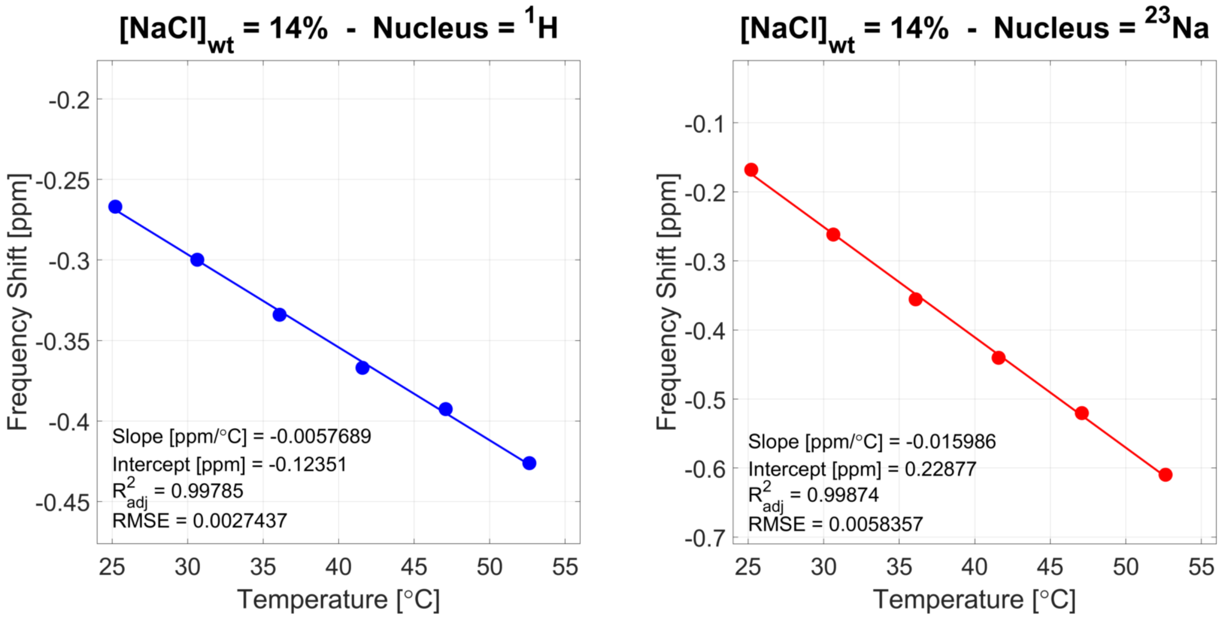

Supplementary Figure 7. Proton (^{1}H) and sodium (^{23}Na) spectra frequency shifts at different temperatures for solution with [NaCl] = 14% weight.

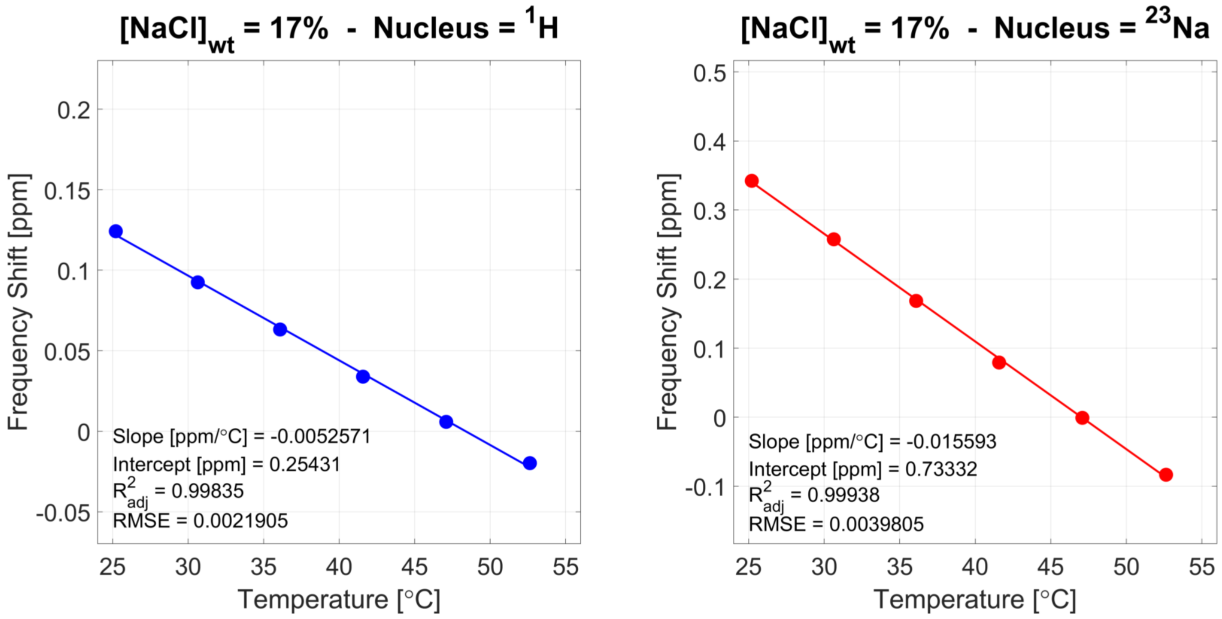

Supplementary Figure 8. Proton (^{1}H) and sodium (^{23}Na) spectra frequency shifts at different temperatures for solution with [NaCl] = 17% weight.

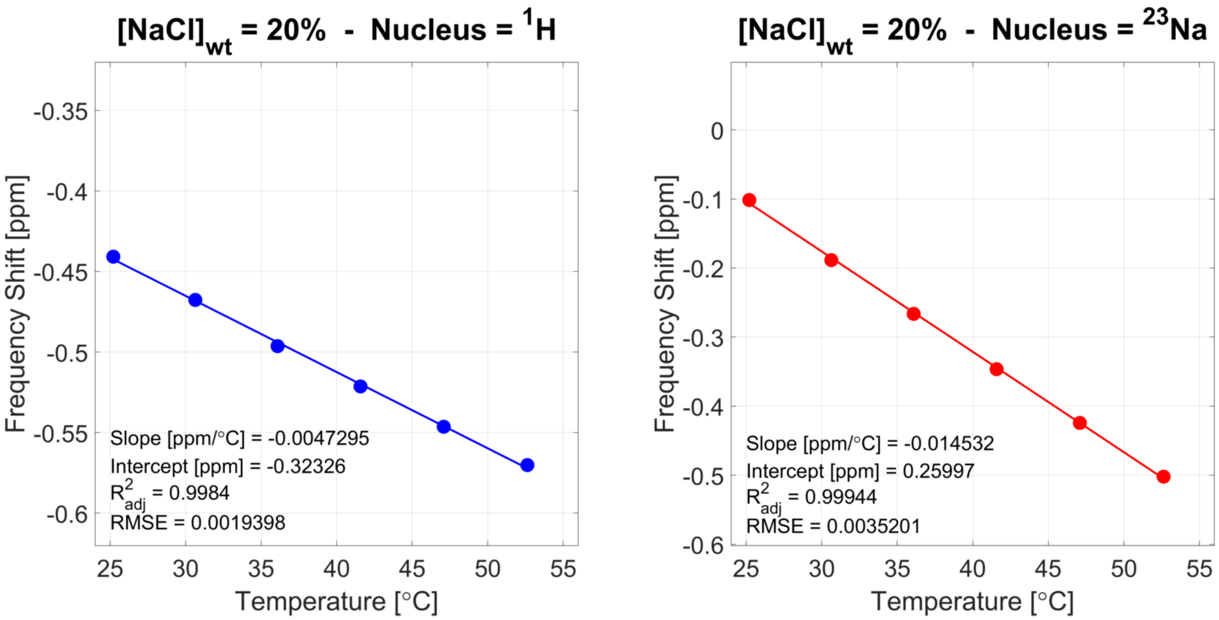

Supplementary Figure 9. Proton (^{1}H) and sodium (^{23}Na) spectra frequency shifts at different temperatures for solution with [NaCl] = 20% weight.

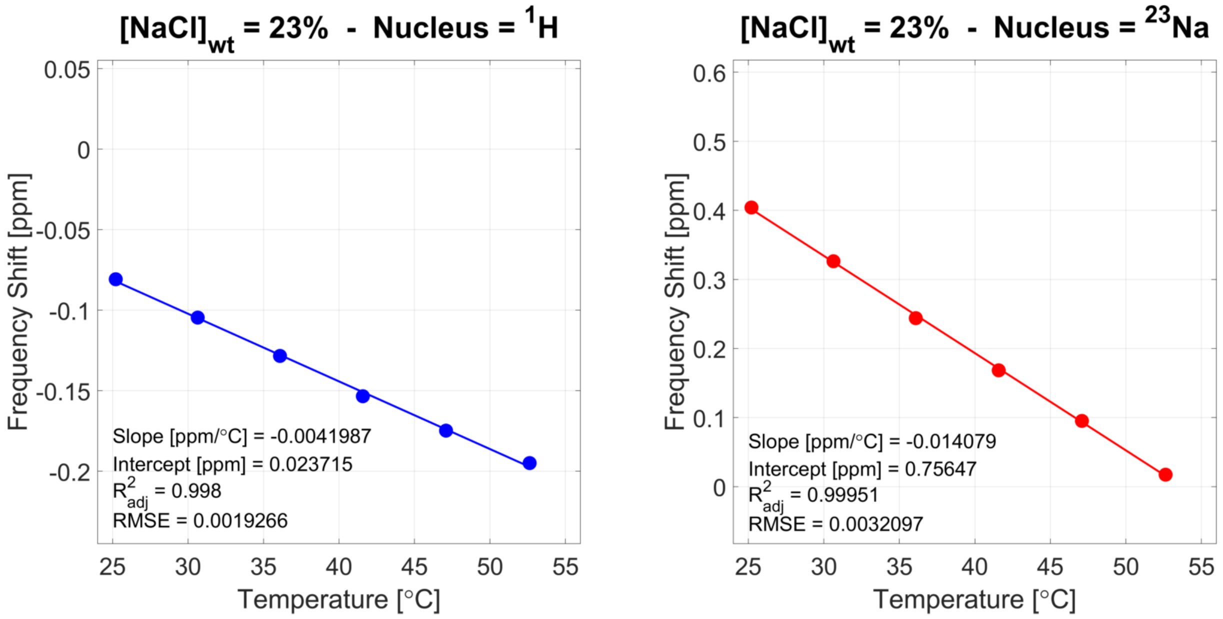

Supplementary Figure 10. Proton (^1H) and sodium (^{23}Na) spectra frequency shifts at different temperatures for solution with [NaCl] = 23% weight.

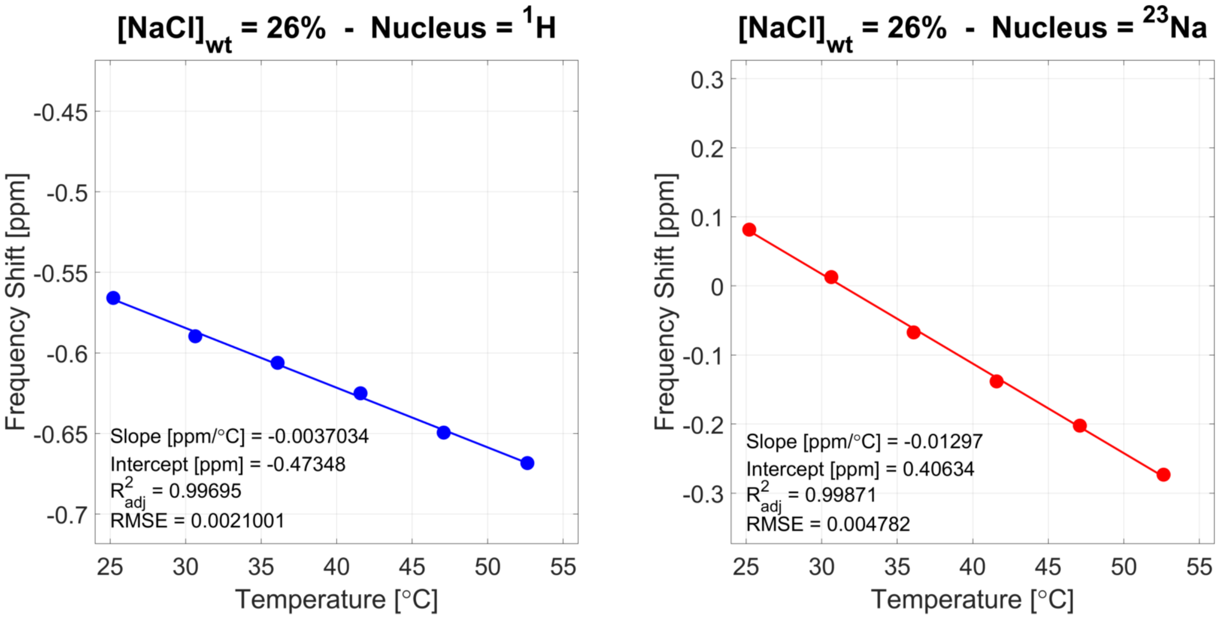

Supplementary Figure 11. Proton (^{1}H) and sodium (^{23}Na) spectra frequency shifts at different temperatures for solution with [NaCl] = 26% weight.

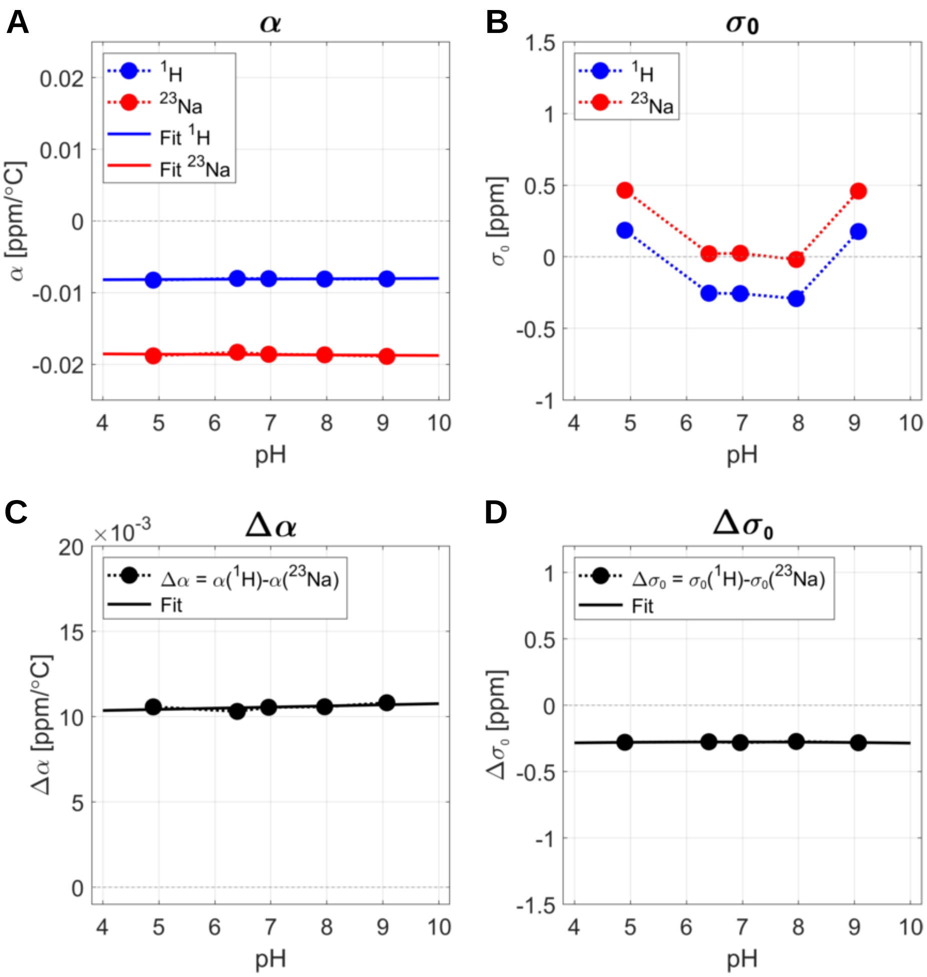

Supplementary Figure 12. α (ppm/°C), σ_0 (ppm), $\Delta\alpha$ (ppm/°C) and $\Delta\sigma_0$ (ppm) values for ^1H and ^{23}Na in a solution with 1% NaCl, for different pH.

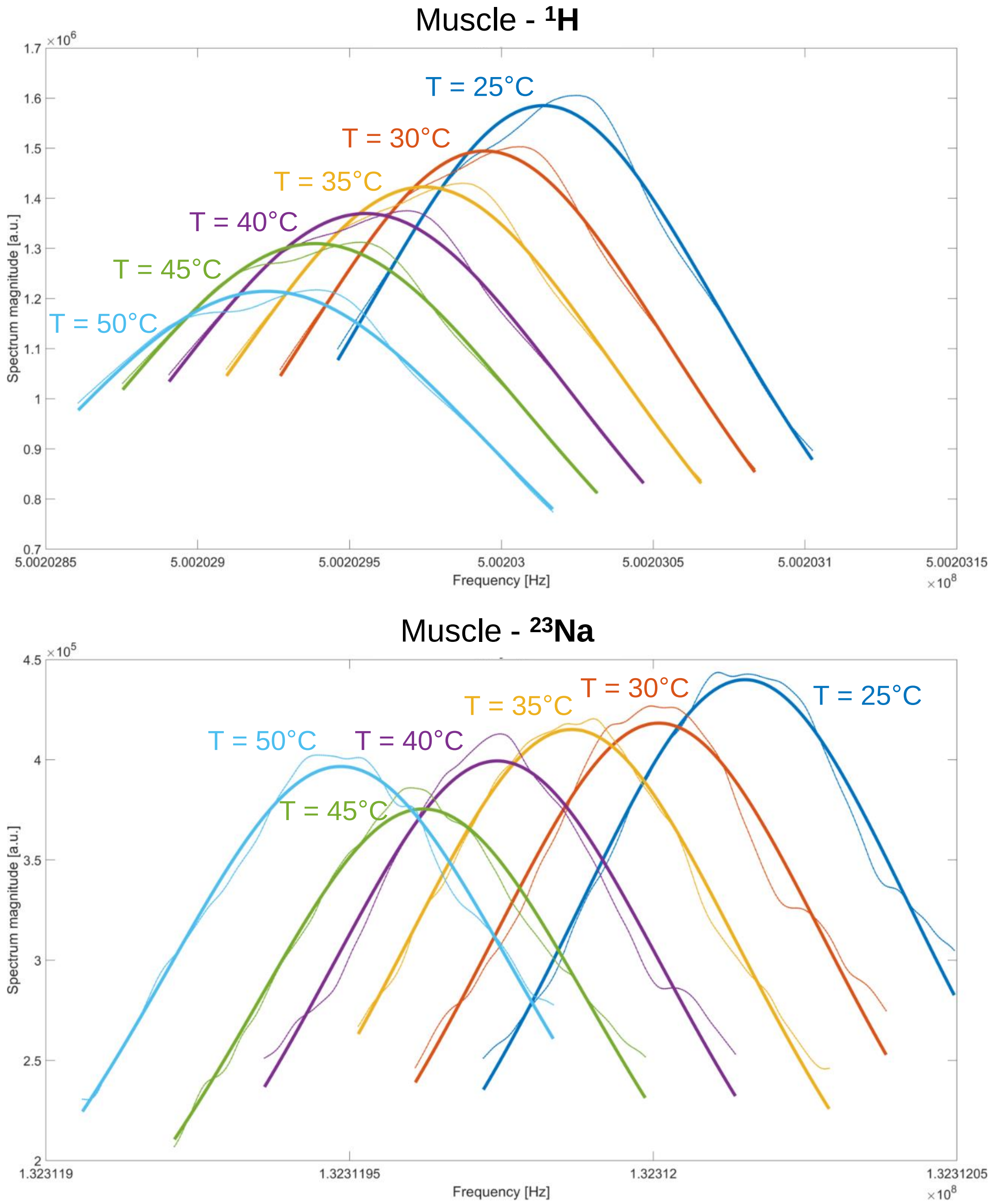

Supplementary Figure 13. Examples of single Lorentzian fitting of 256+1 data points around the maximum of the ^{1}H and ^{23}Na spectra at different temperatures for the muscle tissue sample. Fit curves appear as solid (thick) lines, original data appears as linked data points (thin lines).

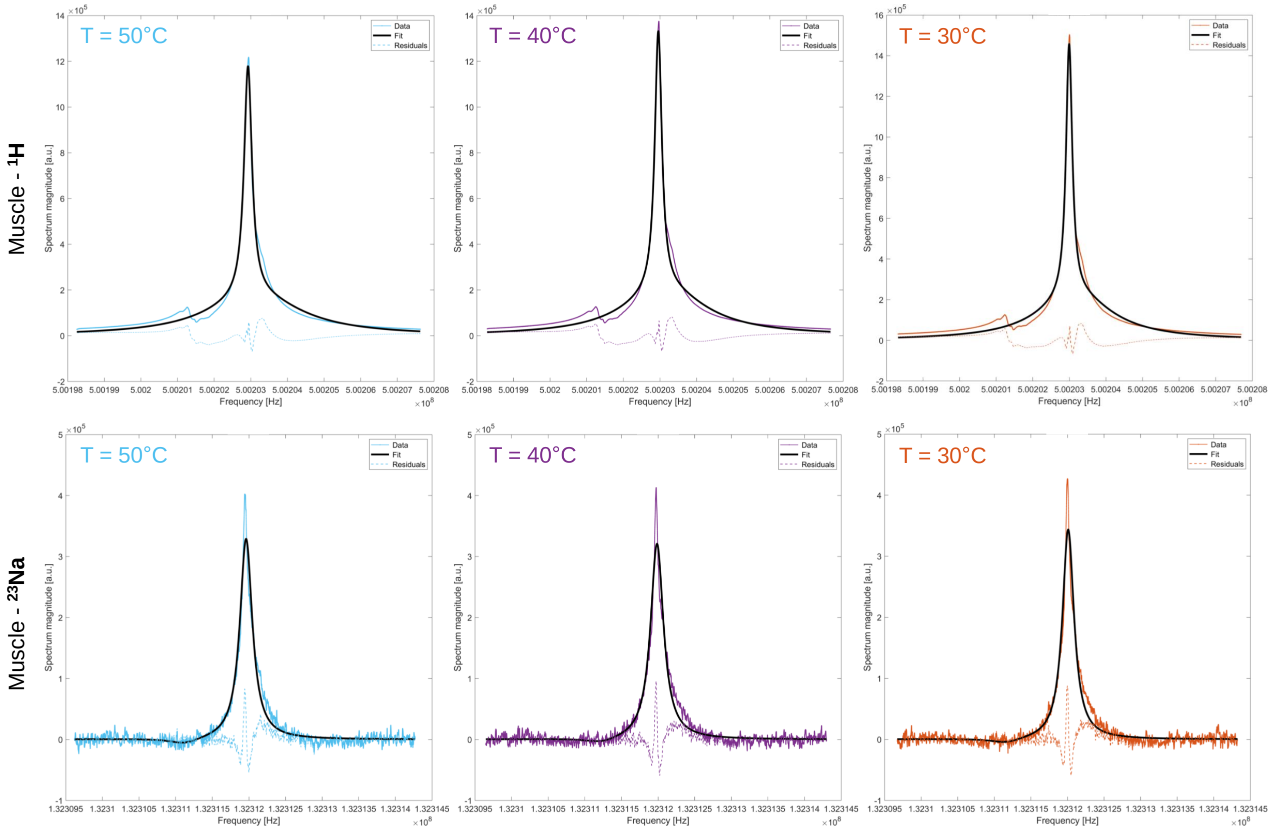

Supplementary Figure 14. Examples of bi-Lorentzian fitting of full ^{1}H and ^{23}Na spectra at 3 different temperatures for the muscle tissue sample. Fit curves appear as black solid lines, original data appears as linked data points (colored lines), and residuals between data and fit appear in dashed colored lines.

Supplementary Table 1. Spectrometer temperature calibration.

Temperature (°C)						
Theoretical (spectrometer)	25	30	35	40	45	50
Corrected (real)	25.21	30.64	36.09	41.58	47.09	52.63

Supplementary Table 2. NaCl concentration calibration.

[NaCl]											
$C_{\%wt}$ (% weight)	0.1	1	2	5	8	11	14	17	20	23	26
$\sigma_{C_{\%wt}}$ (% weight)	±0.05	±0.5	±0.5	±0.5	±0.5	±0.5	±0.5	±0.5	±0.5	±0.5	±0.5
$C_{mol/L}$ (mol/L)	0.017	0.172	0.346	0.885	1.446	2.030	2.637	3.269	3.924	4.605	5.311
$\sigma_{C_{mol/L}}$ (mol/L)	±0.008	±0.087	±0.088	±0.091	±0.095	±0.099	±0.103	±0.107	±0.111	±0.115	±0.120

Supplementary Table 3. Linear fits of frequency shifts f vs. temperature T: f = αT+σ_0.

[NaCl] (%wt)	α (ppm/$^\circ$C)	95% CB for α	σ_0 (ppm)	95% CB for σ_0	R^2_{adj}	RMSE
^{1}H						
0.1	-0.008140	(-0.008575,-0.007749)	0.3878	(0.3721,0.4051)	0.99863	0.00310
1	-0.007981	(-0.008452,-0.007467)	0.1943	(0.1739,0.2133)	0.99810	0.00357
2	-0.007882	(-0.008271,-0.007382)	0.2111	(0.1915,0.2271)	0.99779	0.00380
5	-0.007351	(-0.007730,-0.006855)	0.1215	(0.1019,0.1368)	0.99768	0.00364
8	-0.006942	(-0.007342,-0.006576)	-0.1868	(-0.2015,-0.1709)	0.99839	0.00286
11	-0.006271	(-0.006487,-0.006050)	-0.1125	(-0.1213,-0.1038)	0.99887	0.00216
14	-0.005769	(-0.006041,-0.005451)	-0.1235	(-0.1363,-0.1127)	0.99785	0.00274
17	-0.005257	(-0.005507,-0.005008)	0.2543	(0.2442,0.2641)	0.99835	0.00219
20	-0.004729	(-0.004958,-0.004490)	-0.3234	(-0.3330,-0.3143)	0.99840	0.00194
23	-0.004199	(-0.004528,-0.003952)	0.0237	(0.0137,0.0367)	0.99800	0.00193
26	-0.003703	(-0.003924,-0.003423)	-0.4735	(-0.4846,-0.4646)	0.99695	0.00210
^{23}Na						
0.1	-0.018824	(-0.019000,-0.018580)	0.2972	(0.2873,0.3041)	0.99987	0.00221
1	-0.018681	(-0.019180,-0.018150)	0.1398	(0.1189,0.1599)	0.99945	0.00449
2	-0.018311	(-0.018800,-0.017850)	0.1777	(0.1590,0.1970)	0.99958	0.00385
5	-0.017871	(-0.018270,-0.017430)	0.1899	(0.1724,0.2057)	0.99970	0.00319
8	-0.017464	(-0.018300,-0.016640)	-0.0257	(-0.0587,0.0078)	0.99835	0.00729
11	-0.016463	(-0.017110,-0.015770)	0.1382	(0.1107,0.1643)	0.99912	0.00501
14	-0.015986	(-0.016640,-0.015320)	0.2288	(0.2018,0.2544)	0.99874	0.00584
17	-0.015593	(-0.016100,-0.015130)	0.7333	(0.7150,0.7537)	0.99938	0.00398
20	-0.014532	(-0.014980,-0.014110)	0.2600	(0.2433,0.2784)	0.99944	0.00352
23	-0.014079	(-0.014500,-0.013630)	0.7565	(0.7383,0.7730)	0.99951	0.00321
26	-0.012970	(-0.013550,-0.012400)	0.4063	(0.3839,0.4297)	0.99871	0.00478

Abbreviations: CB = Confidence Bounds; RMSE = Root Mean Square Error; R^2_{adj} = adjusted R^2.

Supplementary Table 4. Fitting parameters for α, $\Delta\alpha$, $\Delta\sigma_0$ versus NaCl concentrations in weight % ($C_{\%wt}$), corresponding to Eq. 6 and 7.

Fit parameters	α (ppm/°C) for ^{1}H	α (ppm/°C) for ^{23}Na	$\Delta\alpha$ (ppm/°C)	$\Delta\sigma_0$ (ppm)
a	0.000172	0.000215	-4.303e-05	-0.0004218
95% CB for a	(0.000168,0.000177)	(0.000199,0.000232)	(-5.735$\times 10^{-5}$,-2.87$\times 10^{-5}$)	(-0.0005425,-0.000301)
b	-0.008183	-0.01892	0.01073	-0.02573
95% CB for b	(-0.008248,-0.008117)	(-0.01915,-0.01868)	(0.01053,0.01094)	(-0.02884,-0.02262)
c				0.0829
95% CB for c				(0.06756,0.09823)
R^2_{adj}	0.99866	0.98882	0.81874	0.99898
RMSE	5.77$\times 10^{-5}$	2.09$\times 10^{-4}$	1.83$\times 10^{-4}$	0.01056

Abbreviations: CB = Confidence Bounds; RMSE = Root Mean Square Error; R^2_{adj} = adjusted R^2.

Supplementary References

1. Ammann, C., Meier, P. & Merbach, A. A simple multinuclear NMR thermometer. *Journal of Magnetic Resonance (1969)* **46**, 319–321 (1982).
2. Lide, D. *CRC Handbook of Chemistry and Physics* (CRC Press, Boca Raton, Florida, USA, 2005).
3. Ku, H. Notes on the use of propagation of error formulas. *Journal of Research of the National Bureau of Standards* **70**, 263–273 (1966).
